# Study protocol: Fecal Microbiota Transplant combined with Atezolizumab/Bevacizumab in Patients with Hepatocellular Carcinoma who failed to achieve or maintain objective response to Atezolizumab/Bevacizumab – the FAB-HCC pilot study

**DOI:** 10.1371/journal.pone.0321189

**Published:** 2025-04-15

**Authors:** Katharina Pomej, Adrian Frick, Bernhard Scheiner, Lorenz Balcar, Larissa Pajancic, Anton Klotz, Abelina Kreuter, Katharina Lampichler, Katharina Regnat, Kerstin Zinober, Michael Trauner, Dietmar Tamandl, Christoph Gasche, Matthias Pinter

**Affiliations:** 1 Division of Gastroenterology and Hepatology, Department of Medicine III, Medical University of Vienna, Vienna, Austria; 2 Division of Gastroenterology and Hepatology, Department of Medicine III, Vienna Liver Cancer Study Group, Medical University of Vienna, Vienna, Austria; 3 Department of Biomedical Imaging and Image-guided Therapy, Medical University of Vienna, Vienna, Austria; Kaohsiung Medical University Hospital, TAIWAN

## Abstract

**Background:**

The gut microbiota is often altered in chronic liver diseases and hepatocellular carcinoma (HCC), and increasing evidence suggests that it may influence response to cancer immunotherapy. Strategies to modulate the gut microbiome (i.e., fecal microbiota transplant (FMT)) may help to improve efficacy of immune checkpoint inhibitors (ICIs) or even overcome resistance to ICIs. Here, we describe the design and rationale of FAB-HCC, a single-center, single-arm, phase II pilot study to assess safety, feasibility, and efficacy of FMT from patients with HCC who responded to PD-(L)1-based immunotherapy or from healthy donors to patients with HCC who failed to achieve or maintain a response to atezolizumab plus bevacizumab.

**Methods:**

In this single-center, single-arm, phase II pilot study (ClinicalTrials.gov identifier: NCT05750030), we plan to include 12 patients with advanced HCC who failed to achieve or maintain a response to atezolizumab/bevacizumab. Patients will receive a single FMT via colonoscopy from donors with HCC who responded to PD-(L)1-based immunotherapy or from healthy individuals, followed by atezolizumab/bevacizumab every 3 weeks. The primary endpoint is safety, measured by incidence and severity of treatment-related adverse events. The main secondary endpoint is efficacy, as assessed by best radiological response according to RECISTv1.1 and mRECIST. Additional exploratory endpoints include data on the effect of FMT on recipient gut microbiota, as well as metagenomic analysis of stool samples, analyses of circulating immune cells and serum and stool proteomic, metabolomic and lipidomic signatures.

**Discussion:**

The results of this study will help to define the potential of FMT as add-on intervention in the systemic treatment of advanced HCC, with the potential to improve efficacy of immunotherapy or even overcome resistance.

**Trial registration:**

EudraCT Number: 2022-000234-42

Clinical trial registry & ID: ClinicalTrials.gov identifier: NCT05750030 (Registration date: 16.01.2023)

## Background

Hepatocellular carcinoma (HCC) represents 90% of all primary liver cancers and one of the most common cause of cancer-related death globally [1]. Resection, local ablation, and liver transplantation are potential curative therapies, but are reserved for early-stage HCC. Unfortunately, most patients are diagnosed at an advanced tumor stage, where only palliative treatment options are available. Patients with liver-limited, multifocal HCC are usually treated with transarterial chemoembolization (TACE), while those with macrovascular tumor invasion or extrahepatic metastases are typical candidates for systemic therapy [1].

Encouraging data on immune checkpoint inhibitors (ICIs) in HCC from phase II trials directed the research progressively towards immunotherapy. However, subsequent randomized controlled phase III trials testing programmed cell death protein 1 (PD-1)-targeted monotherapy with nivolumab or pembrolizumab failed to significantly improve survival endpoints [2]. These setbacks suggested that combined systemic approaches may be needed to implement immunotherapy in HCC. VEGF’s promotion of immunosuppression in the tumor microenvironment was the rationale to combine ICIs with VEGF-targeted agents [2]. In a pivotal phase III trial, the combination of atezolizumab plus bevacizumab improved both primary endpoints overall survival (OS) and progression-free survival (PFS) versus sorafenib, and demonstrated good safety and improved quality of life data [3]. Consequently, atezolizumab plus bevacizumab was implemented as the new standard of care in systemic front-line treatment of HCC for most patients [4,5]. Moreover, the combination of tremelimumab plus durvalumab also improved its primary endpoint of OS in a phase III trial [6], and represents an alternative immunotherapy-based treatment option in the systemic first-line setting [7].

Even though these results represent milestones in the systemic treatment of HCC, only around one-third of patients responds to atezolizumab/bevacizumab [3]. While patients with stable disease (SD) still show improved overall survival compared to subjects with progressive disease (PD), patients with complete (CR) or partial response (PR) are those most likely to derive a long-term survival benefit from immunotherapy [8]. Currently, there is no established biomarker to predict response to immunotherapy [2,9].

The gut microbiota is often altered in chronic liver diseases and HCC and may modulate cancer-promoting and cancer-suppressing pathways associated with immunity and inflammation [10]. For instance, in metabolic dysfunction-associated steatohepatitis (MASH)-related HCC, gut dysbiosis promotes peripheral immunosuppression [11]. Increasing evidence suggests that the gut microbiome may influence response to cancer immunotherapy [12–14]. Hence, strategies to modulate the gut microbiome (i.e., FMT) may help to improve efficacy of ICIs or even overcome resistance to immunotherapy. Indeed, in preclinical studies, FMT obtained from patients responding to ICIs into mice enhanced the efficacy of anti-PD-(L)1 treatment and augmented T cell response, while FMT from non-responders did not [12–14]. Two recently published clinical pilot studies confirmed the feasibility and safety of this innovative approach in humans [15,16]. In a phase I clinical trial, 10 metastatic melanoma patients with confirmed progression on PD-1-targeted immunotherapy received FMT from donors with metastatic melanoma who had achieved complete remission for at least 12 months with PD-1-targeted immunotherapy. Reintroduction of anti-PD-1 treatment after FMT led to 2 partial responses and 1 complete response. The safety profile was excellent with mild bloating being the only FMT-related adverse event [15]. The second clinical trial investigated the safety and efficacy of responder-derived FMT in combination with PD-1-targeted immunotherapy in patients with melanoma primary refractory to anti-PD-1 treatment. Of 15 patients evaluable for radiological response, 3 achieved complete (n=1) or partial (n=2) remission and 3 patients had durable stable disease for more than 12 months. No relevant FMT-related adverse events were reported [16]. Moreover, a recently published phase I trial testing upfront combination of healthy donor FMT with first-line immunotherapy in 20 advanced melanoma patients reported a good safety profile and an encouraging objective response rate of 65% (including four complete responses) [17].

Here, we describe the design and the rationale of FAB-HCC, a single-center, single-arm, phase II pilot study of atezolizumab plus bevacizumab in combination with FMT in patients with HCC who failed to achieve or maintain a response to atezolizumab plus bevacizumab. The primary endpoint of this study is safety (i.e., incidence and severity of treatment-related adverse events), and secondary endpoints include radiological response, progression-free and overall survival, as well as quality of life.

## Methods and study design

FAB-HCC is a single-center, single-arm, phase II pilot study designed to evaluate the safety, feasibility, and efficacy of atezolizumab plus bevacizumab in combination with FMT from anti-PD-(L)1 responders or healthy donors to adult patients with HCC who failed to achieve or maintain a complete or partial radiological response to atezolizumab plus bevacizumab according to mRECIST [18].

The study was approved by the Ethics Committee of the Medical University of Vienna (Date of approval: 27.05.2022, approval number: 1054/2022), as well as the Austrian Competent Authorities (Bundesamt für Sicherheit im Gesundheitswesen (BASG) represented by the Agency for Health and Food Safety (AGES Medizinmarktaufsicht)). If necessary, amendments to the protocol are possible and must be disclosed to the respective authorities. The clinical trial will be performed in full compliance with the legal regulations according to the Drug Law (AMG – Arzneimittelgesetz) of the Republic of Austria and was registered to the European Clinical Trial Database (EudraCT number: 2022-000234-42). The study will be performed in compliance with the Declaration of Helsinki and the principles of Good Clinical Practice. Written/signed informed consent is obtained from each patient and donor before study entry.

The study is planned to enroll a total of 12 patients with HCC, mainly patients who progressed according to mRECIST on this treatment, but study participation will also be offered to patients who have achieved stable disease as best radiological response according to mRECIST after the first 12 months of treatment initiation, as it is unlikely that a response to atezolizumab/bevacizumab will occur after one year [19]. Eligible patients will receive one single FMT on Day 0, followed by Cycle 1 of atezolizumab/bevacizumab (± 3 days), which they will continue to receive at the approved standard dose every 3 weeks (± 5 days) thereafter.

The investigational medicinal products (IMPs) used in this pilot study are atezolizumab and bevacizumab. Atezolizumab (trade name: Tecentriq, manufacturer: Roche), is an Fc-engineered, humanized IgG1 anti-programmed death 1 ligand 1 (PD-L1) monoclonal antibody produced in Chinese hamster ovary cells by recombinant DNA technology [20]. Atezolizumab will be administered by IV infusion at a fixed dose of 1200 mg on day 1 and then every 21 days. Bevacizumab (trade name: Avastin, manufacturer: Roche), is a recombinant humanized monoclonal antibody against VEGF and is also produced by DNA technology in Chinese Hamster Ovary cells. Bevacizumab will be administered by IV infusion at a dose of 15mg/kg on day 1 and then every 21 days. The clinical safety profile for the combination of atezolizumab plus bevacizumab as therapy in HCC has emerged from clinical trials [3].

### Eligibility criteria

FAB-HCC will recruit patients (aged ≥18 years) with histologically or radiologically confirmed HCC with progressive disease (according to mRECIST) during treatment with atezolizumab/bevacizumab (without or with prior complete or partial response as best radiological response according to mRECIST) or patients with stable disease as best radiological response (according to mRECIST) after the first 12 months of atezolizumab/bevacizumab treatment. Patients will require adequate hematological and end-organ functions, as well as a known variceal status with adequate medical or endoscopic treatment. Detailed eligibility criteria are provided in Table 1.

**Table 1:** Eligibility criteria of FAB-HCC study.

Key inclusion criteria	Key exclusion criteria
Signed informed consent form	Known fibrolamellar carcinoma or mixed cholangiocellular carcinoma
Age ≥ 18 years	Massive tumor progression (>100% increase in target lesions or progression associated with significant clinical deterioration)
Histologically or radiologically confirmed HCC	Uncontrolled ascites
Patients with progressive disease (according to mRECIST) during treatment with atezolizumab/bevacizumab (without or with prior complete or partial response as best radiological response according to mRECIST) OR patients with stable disease as best radiological response (according to mRECIST) after the first 12 months of atezolizumab/bevacizumab treatment	Overt hepatic encephalopathy or concomitant treatment with rifaximin
Negative HIV test	Prior allogeneic stem cell or solid organ transplantation
Patients with chronic hepatitis B must be under antiviral treatment and hepatitis B DNA must be <500 IU/mL	Active or history of severe autoimmune disease
Variceal status must be known and if present, adequate medical or endoscopic treatment is required	History of idiopathic pulmonary fibrosis, organizing pneumonia (e.g., bronchiolitis obliterans), drug-induced pneumonitis, or idiopathic pneumonitis, or evidence of active pneumonitis
ECOG Performance Status 0–1	Significant cardiovascular disease (such as New York Heart Association Class II or greater cardiac disease, myocardial infarction, or cerebrovascular accident) within 3 months prior to study inclusion or unstable angina
Child-Pugh class A-B8	Severe infection within 4 weeks prior to study inclusion
Adequate hematological and end-organ function, defined as follows: - AST and ALT < 10 x ULN - Serum bilirubin <3.5 mg/dL - Albumin ≥28 g/L - Serum creatinine ≤ 1.5 mg/dL - Hemoglobin ≥ 8 mg/dL - Platelet count ≥ 50 G/L - Leukocytes ≥ 2.5 G/L - Patients not receiving therapeutic anticoagulation: INR ≤ 2.3 or thromboplastin time ≥ 40%	Treatment with systemic immunosuppressive medication with the following exceptions: - Acute, low-dose systemic immunosuppressant medication or a one-time pulse dose of systemic immunosuppressant medication (e.g., 48 hours of corticosteroids for contrast allergy) - Mineralocorticoids (e.g., fludrocortisone), corticosteroids for chronic obstructive pulmonary disease or asthma, or low-dose corticosteroids for adrenal insufficiency
Women of childbearing potential must agree to remain abstinent (refrain from heterosexual intercourse) or use contraceptive methods	Pregnant or breastfeeding women
Men must agree to remain abstinent (refrain from heterosexual intercourse) or use a condom	Significant vascular disease (e.g., peripheral arterial thrombosis) within 6 months prior to study inclusion
	Major surgery within 4 weeks prior to study inclusion or minor surgery (excluding placement of a vascular access device) within 3 days prior to study inclusion
	History of gastrointestinal fistula or perforation, or intraabdominal abscess within 6 months prior to study inclusion
	Serious, non-healing wound or active ulcer

Abbreviations: ALT, alanine aminotransferase; AST, aspartate aminotransferase; DNA, deoxyribonucleic acid; ECOG PS, Eastern Cooperative Oncology Group performance status; HCC, hepatocellular carcinoma; HIV, human immunodeficiency virus; INR, international normalized ratio; mRECIST, modified Response Evaluation Criteria in Solid Tumors; RECISTv1.1, Response Evaluation Criteria in Solid Tumors version 1.1.

### Study period and planned sample size

The start of the study is defined as the date of the first patient’s first visit. The end of the study is defined as the date when every patient enrolled had a follow-up of at least 20 weeks (2 scheduled follow-up imaging visits), if not progressed or deceased earlier. Based on previous phase III second-line studies in HCC [21–23], a disease control rate of less than 50% is below what would be expected. Thus, to ensure that a likely unsuccessful therapeutic strategy is not pursued, the study will stop enrollment prematurely if the disease control rate of the first 6 patients included is less than 50%.

This clinical trial plans to include 12 patients. The start of recruitment period for this study is planned for August 14, 2023 and the end of recruitment period is planned for May 31, 2025. The number of patients planned to be included is expected to be sufficient to fulfill the pilot study’s objectives. No inferential statistical testing is planned for the primary safety endpoint and secondary efficacy endpoints, and therefore an exact sample size requirement has not been specified. Previous studies evaluating the efficacy of FMT in patients with advanced melanoma not responding to immunotherapy included a comparable number of patients [15,16].

### Study procedures

**Fig 1:**
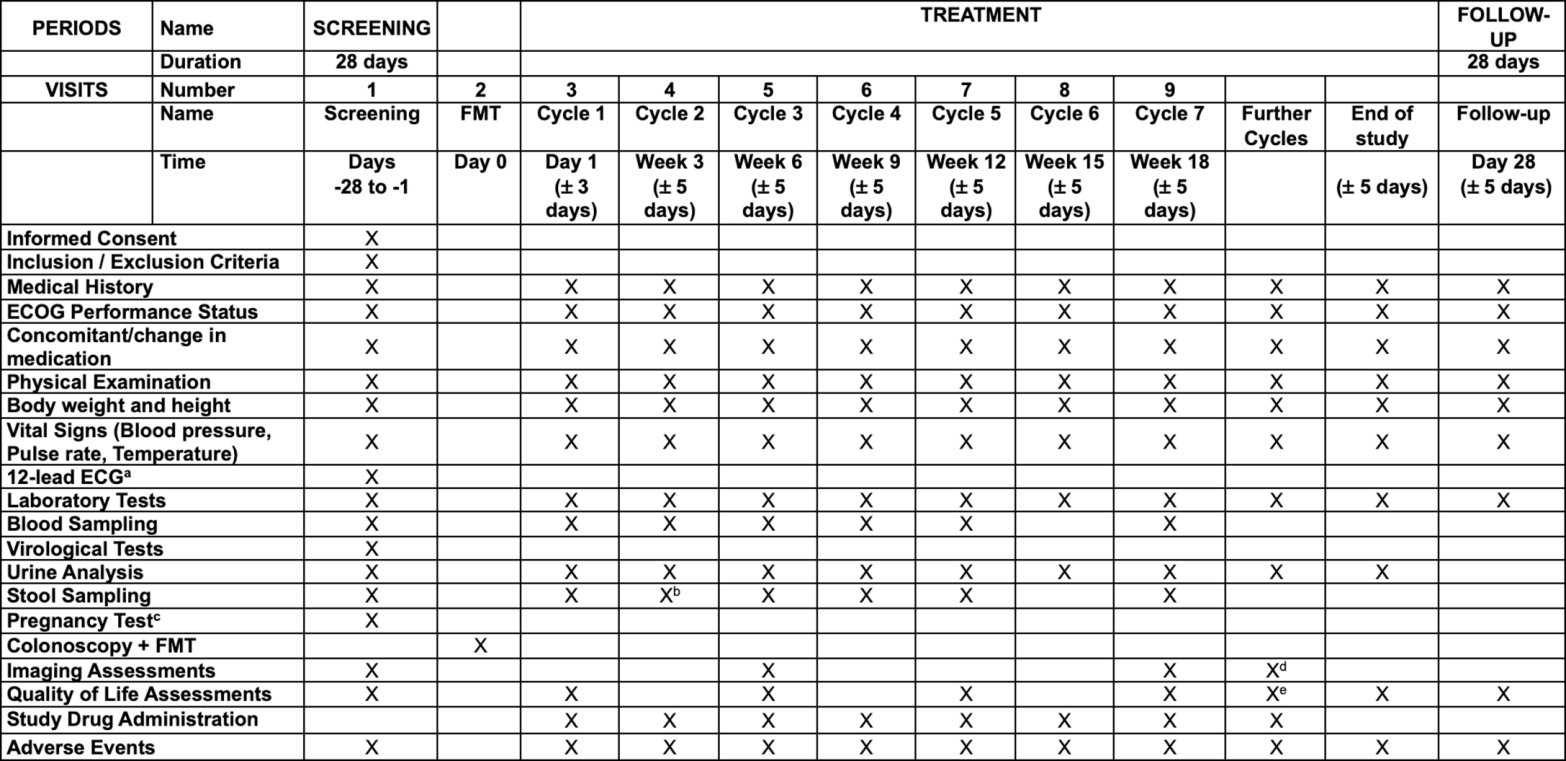
SPIRIT schedule of enrollment. ^a^12-lead echocardiography (ECG) during screening and on Day 1. Further ECGs should be performed upon clinical suspicion. ^b^At week 1, week 2, and week 3. ^c^Repetitive pregnancy assessment in women with childbearing potential. ^d^Every 4^th^ cycle. ^e^Every other visit (odd cycles).

**Fig 2:**
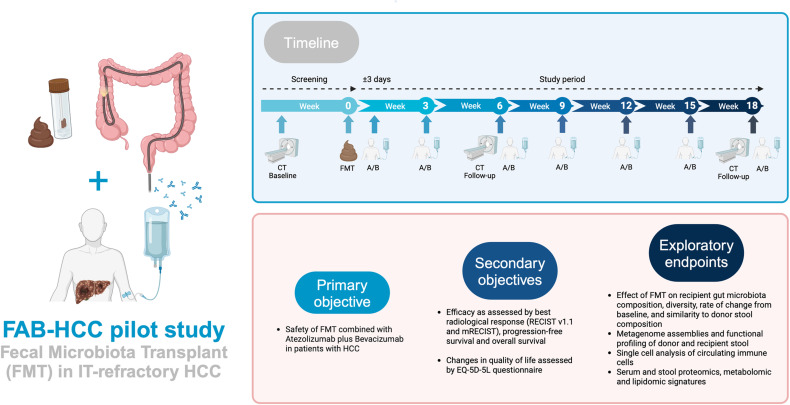
FAB-HCC study flow chart. (original figure, no copyright permission required, created with BioRender.com), Abbreviations: A/B, atezolizumab plus bevacizumab; CT, computed tomography; FMT, fecal microbiota transplant; HCC, hepatocellular carcinoma; IT, immunotherapy; mRECIST, modified Response Evaluation Criteria in Solid Tumors; RECISTv1.1, Response Evaluation Criteria in Solid Tumors version 1.1.

After obtaining written informed consent, screening evaluations must be completed and reviewed by study staff to confirm that patients meet all eligibility criteria before enrollment. All necessary study procedures (including medical history, concomitant medication, physical examination, vital signs, and electrocardiogram) will be performed at baseline and prespecified visits. The schedule of activities to be performed and a study flow chart are provided in Figs 1 and 2. Laboratory testing will be performed at every visit, and blood and stool sampling will take place at predefined timepoints (see Fig 1 and S1 Table). Patients included in the FAB-HCC trial, will undergo tumor imaging at week 6 and every 12 weeks thereafter. FMT will be performed once on Day 0. Patient-reported outcome data will be obtained using the EQ-5D-5L questionnaire to fully characterize the study participants.

### FMT: donor selection, preparation and storage of fecal material for FMT, patient preparation and application of fecal material by FMT

FMT is still a novel treatment modality and therefore, data about the most efficient way of performing FMT is scarce. Our study will be conducted according to the Austrian consensus [24] as well as the European guidelines on FMT [25].

Potential donors will be patients with unresectable HCC treated with anti-PD-(L)1-based immunotherapy with complete or partial response for at least 12 months or healthy donors. In total, we are planning to include 2 donors, one immunotherapy responder and one healthy donor, of whom stool for FMT will be used for 6 patients each, after ruling out infectious agents in the stool by repetitive stool sampling. Donor-specific exclusion criteria are listed in Table 2. Initial serological testing will be performed within 14 days prior to donor stool acquisition. Processed donor stool will be tested for the respective targets by applying the methods listed in Table 3. Donors repeatedly used for FMT will be retested every 6 months or sooner, in case of certain risks for infectious diseases [16].

**Table 2:** Donor-specific exclusion criteria.

History of antibiotic treatment within 2 months preceding donation
History of intrinsic gastrointestinal illnesses including inflammatory bowel disease, irritable bowel syndrome, chronic diarrhea (i.e., celiac disease), active primary gastrointestinal malignancies, or major gastrointestinal surgical procedures
History of symptomatic autoimmune illness
History of documented chronic pain syndromes (fibromyalgia, chronic fatigue) or neurologic and neurodevelopmental disorders
History of metabolic syndrome, severe obesity (body mass index > 35), or moderate-to-severe malnutrition (as assessed clinically)
Donors with positive tests for SARS-CoV-2, latent potential (human immunodeficiency virus, hepatitis B virus, hepatitis C virus [only if PCR positive], human T-cell lymphotropic virus type 1 [HTLV-1], HTLV-2, strongyloides, syphilis) and/or had evidence of multi-drug resistant organisms such as vancomycin- resistant *Enterococcus*, carbapenem-resistant *Enterobacteriaceae*, and extended spectrum beta-lactamase are ineligible
FMT-relevant severe immune-related adverse events, including colitis and hepatitis

Abbreviations: FMT, fecal microbiota transplant; HTLV, human T-cell lymphotropic virus; PCR, polymerase chain reaction; SARS-CoV-2, severe acute respiratory syndrome coronavirus type 2.

**Table 3:** Targets and tests for processed donor stool analyses.

	Target	Test
**Bacteria**	ESBL, VRE, multidrugresistant gramnegative rods	Stool culture
	Clostridioides difficile (+toxin)	Stool culture, ELISA
	EAEC, EPEC, ETEC, EHEC, EIEC, tox C.diff, Campylobacter spp, Plesiomonas shigelloides, Salmonella spp., Vibrio parahaemolyticus, Vibrio vulnificus, Vibrio cholerae, Yersinia enteroloytica	Stool multiplex PCR
	Treponema pallidum	Serologic TPPA, TPHA
	Tbc	Serologic Quantiferon
**Parasites**	Cryptosporidium spp, Cyclospora cayetanensis, Entamoeba histolytica, Giardia lamblia	Stool multiplex PCR, antigen detection
	Ascariosis, Echinococcosis, Fasciolosis, Strongyloidosis, Toxocariosis, Trichinellosis, Cysticercosis, Trypanosoma cruzei/brucei	Serological testing
	Worms, Microspora, Protozoa, Helminths, Entamoeba histolytica, Lamblia giardia, Cryptospora, Cyclospora, Isospora	Stool microscopy, SAF fixation, antigen detection, Ziehl-Neelsen stain
**Viruses**	Adenovirus F40/41; Astrovirus, Norovirus GI/GII, Rotavirus A; Sapovirus (I/II/IV/V)	Stool multiplex PCR
	Hepatitis A, B, C, D, E; HIV; CMV, HSV1+2, EBV, VZV, HHV6, HTLV 1+2; JC-Virus	Serological testing, PCR, Antibody detection

Abbreviations: CMV, cytomegalovirus; EAEC, enteroaggregative Escherichia coli; EBV, Epstein-Barr virus; EHEC, enterohemorrhagic Escherichia coli; EIEC, enteroinvasive Escherichia coli; ELISA, enzyme-linked immunosorbent assay; EPEC, enteropathogenic Escherichia coli; ESBL, extended-spectrum β-lactamase; ETEC, enterotoxigenic Escherichia coli; HIV, human immunodeficiency virus; HHV6, human herpes virus 6; HSV, herpes simplex virus; HTLV, human T-Lymphotropic virus; JC-virus, John Cunningham virus; PCR, polymerase chain reaction; SAF, sodium acetate-acetic acid-formalin; Tbc, Mycobacterium tuberculosis; TPHA, treponema pallidum hemagglutination assay; TPPA, treponema pallidum particle agglutination assay; VRE, vancomycin-resistant enterococci; VZV, Varicella zoster virus.

Fecal material will be collected and prepared as described elsewhere [26,27]. Donor feces will be collected after the completion of the safety screening phase. Donor’s feces are processed under good manufacturing practice (GMP) conditions and for the preparation process, regulations for the work with feces, classified as biohazard level 2, will be followed (wearing of water-repellent garments, gloves, facemasks, protective goggles, or shields). After the extraction of the fecal probe (approximately 50g of fecal matter for one colonoscopy-based implant), the entire donation will be diluted with 100–500 mL of sterile saline (0.9% NaCl) using 3x the weight of donor stool. Afterwards the mixture is homogenized in a sterilized blender or similar device, especially designated for this purpose. Afterwards the probe will sequentially be sieved to remove particulate material. Since instant application of fresh fecal probes to study recipients is not possible, the processed donor stool is mixed with glycerol (10% of total weight) and frozen at monitored temperature (-80°C). Before usage, the fecal probes will be gently reheated in a 37°C warm water bath over a period of 2 hours.

Patients will be prepared according to standard care with a colon lavage, routinely given prior to colonoscopy. To deplete the innate gut microbiota, patients receive an antibiotic treatment, according to protocols previously published [15,24,28], prior to colonoscopy consisting of an oral antibiotic treatment with vancomycin 500mg and neomycin 1000mg q6h for 72 hours.

For the application process, the Austrian consensus paper [24] recommends the lower gastrointestinal tract as the preferable route. The colonoscopy will be performed by an experienced endoscopist, and the procedure does not alter from standard colonoscopies, except for the administration of the fecal probes. During bowel intubation with the colonoscope residual stool will be suctioned to achieve maximal mucosal cleansing. After reaching 20 cm into the terminal ileum, a 100mL fecal implant suspension will be administered via a catheter in the following sequence: 20mL in the terminal ileum, 40mL in the cecum and right colon, 20mL in the transverse colon and 20mL in the descending colon. After procedure completion, the recipients remain lying on prone Trendelenburg position for at least 4 hours to maintain the FMT suspension in the bowel. The day of the colonoscopy-based FMT is considered as day 0 of the trial protocol [15]. In patients with insufficient bowel lavage (i.e., patients with significant amounts of residual stool), colonoscopy will be prematurely terminated (due to an increased risk for bowel perforation), bowel lavage will be repeated and a second colonoscopy will be scheduled.

### Outcome measures and primary and secondary endpoints

The primary endpoint will be analyzed using descriptive statistics. In detail, the incidence and severity of treatment-related adverse events determined according to National Cancer Institute (NCI) Common Terminology Criteria for Adverse Events (CTCAE) version 5.0 will be described. Secondary endpoints include efficacy and quality of life. Efficacy will be evaluated by the number (percentage) of study participants achieving complete response (CR), partial response (PR), stable disease (SD) or progressive disease (PD) as best radiological response evaluated according to mRECIST and RECIST v1.1 criteria. Objective response is defined as either complete or partial response, while disease control rate comprises complete/partial response as well as stable disease. Changes of quality of life during the study period as assessed by EQ-5D-5L questionnaire will be evaluated using paired T-test or Wilcoxon signed rank test. Exploratory endpoints will include data on the effect of FMT on recipient gut microbiota, as well as metagenomic analysis of stool samples, single cell analyses of circulating immune cells, and serum and stool proteomic, metabolomic and lipidomic signatures.

### Adverse events management and dose modifications

All adverse events (both non-serious and serious), whether reported by the patient or noted by study personnel, will be classified in associated and not associated with the IMP, and will be recorded. Adverse events associated with IMP exposure and likely to have an immune-mediated underlying mechanism will be considered as adverse events of special interest (AESI). AESI may occur already after the first dose to weeks/months after the last dose. IMP will be interrupted or discontinued, after excluding other potential etiologic causes, if an AESI is suspected. A detailed list of AESIs for this study can be found in Table 4.

**Table 4:** List of adverse events of special interest (AESIs).

Cases of potential drug-induced liver injury that include an elevated ALT or AST (>3 x baseline value) in combination with either an elevated bilirubin (>2 x ULN) or clinical jaundice, as defined by Hy’s Law
Suspected transmission of an infectious agent by a study treatment, as defined below: -Any organism, virus, or infectious particle (e.g., prion protein transmitting transmissible spongiform encephalopathy), pathogenic or non-pathogenic, is considered an infectious agent. A transmission of an infectious agent may be suspected from clinical symptoms or laboratory findings that indicate an infection in a patient exposed to a medicinal product. This term applies only when a contamination of the study treatment is suspected.
Pneumonitis
Colitis
Endocrinopathies (e.g., diabetes mellitus, pancreatitis, adrenal insufficiency, hyperthyroidism, hypophysitis)
Hepatitis, including AST or ALT >10 x ULN
Systemic lupus erythematosus
Neurological disorders (e.g., Guillan-Barré syndrome, myasthenic syndrome or myasthenia gravis, meningoencephalitis)
Events suggestive of hypersensitivity, infusion-related reactions, cytokine release syndrome, HLH, and MAS
Nephritis
Ocular toxicities (e.g., uveitis, retinitis, optic neuritis)
Myositis and myopathies, including rhabdomyolysis
Grade ≥ 2 cardiac disorders (e.g., atrial fibrillation, myocarditis, pericarditis)
Vasculitis
Autoimmune hemolytic anemia
Severe cutaneous reactions (e.g., Stevens-Johnson syndrome, dermatitis bullous, toxic epidermal necrolysis)
Grade ≥3 hypertension
Grade ≥3 proteinuria
Any grade GI perforation, abscess, or fistula
Grade ≥2 non-GI fistula or abscess
Grade ≥3 wound-healing complication
Any arterial thromboembolic event
Hemorrhage (e.g., grade ≥2 hemoptysis, other grade ≥3 hemorrhagic event)
Grade ≥3 venous thromboembolic event
Grade ≥3 chronic heart failure

Abbreviations: ALT, alanine aminotransferase; AST, aspartate aminotransferase; GI, gastrointestinal; HLH, hemophagocytic syndrome; MAS, macrophages activation syndrome; ULN, upper limit of normal.

No dose modifications are allowed for atezolizumab and bevacizumab. However, treatment may be temporarily interrupted in patients experiencing toxicity considered to be related to study treatment. Furthermore, if continued administration of the study drug is believed to be contrary to the best interests of the patient, the investigator must temporarily interrupt or permanently discontinue the study drug. The interruption or premature discontinuation of study drug might be triggered by an AE, a diagnostic or therapeutic procedure, an abnormal assessment (e.g., laboratory abnormalities), or for administrative reasons, in particular withdrawal of the patient’s consent.

### Statistical analysis

For the analysis of the primary safety endpoint, all subjects who underwent technically successful fecal microbiota transplantation and received the study drug (at least one dose) and did not violate the protocol in a way that might affect the evaluation of the effect of the combination of FMT and the study drugs on the primary endpoint, i.e., without major protocol violations, will be included. Patients who do not undergo FMT will be replaced.

An interim analysis of clinical data will be performed after 6 subjects (50% of the planned study population) underwent at least one radiological follow-up. If disease control (CR/PR/SD) as best radiological response according to mRECIST cannot be achieved in ≥50% (3 subjects) of the first 6 participants, the study will be terminated prematurely as a disease control of >50% would be expected with alternative second line treatment options. Full data analysis, including exploratory endpoints, will be performed when the study is completed.

The primary endpoint and secondary efficacy endpoints will be analyzed using descriptive statistics. Kaplan-Meier method will be used to calculate progression-free survival and overall survival. Distribution of quality-of-life parameters will be assessed by plotting histograms. Baseline and follow-up values will be demonstrated as mean (+/- standard deviation) or median (IQR), as applicable. Changes of quality of life during the study period as assessed by EQ-5D-5L questionnaire will be evaluated using paired T-test or Wilcoxon signed rank test.

### Exploratory endpoints

In addition to clinical endpoints, FAB-HCC will collect blood and stool samples at different timepoints (Supplemental Table 1) to investigate different exploratory endpoints and generate mechanistical hypotheses. The effect of FMT on recipient gut microbiota composition, diversity (alpha and beta), rate of change from baseline and similarity (Bray-Curtis dissimilarity) to donor stool composition over time (engraftment) as well as comparison of responders and non-responders, will be analyzed by extracting bacterial DNA from stool by using an established pipeline with MiSeq technology. Metagenome assemblies and functional pathway analysis will be performed by using shotgun metagenomic sequencing. We will also collect peripheral blood mononuclear cells (PBMCs) before and at different timepoints after FMT for single cell profiling. Finally, serum and stool proteomic, metabolomic, and lipidomic signatures before and after FMT will be analyzed after processing data using specific commercial software.

### DISCUSSION

The combination of atezolizumab plus bevacizumab represents a reference standard in systemic front-line therapy for advanced stage HCC [3,4,7]. Patient who respond to this treatment can derive a long-term survival benefit [8]. However, only around one-third of patients treated with atezolizumab/bevacizumab achieves CR or PR, while around 20% have a primary resistance reflected by disease progression already at the first radiological evaluation. Patients who achieve stable disease (~40%) have an initial clinical benefit, but most of them will eventually show progression of the disease during the course of treatment [3].

Disease progression usually triggers a change in systemic treatment, but given that all available second-line agents have been tested in sorafenib-pretreated patients, no established second-line option exists after atezolizumab/bevacizumab and treatment sequencing after first-line immunotherapy remains purely empirical [9]. Approved targeted therapies (i.e., sorafenib, lenvatinib, regorafenib, cabozantinib, ramucirumab) are recommended as per off-label availability [4]. However, these agents have limited efficacy (median OS improvement: <3 months vs. placebo; response rate: 2%-11%) [21,22,29]. Only lenvatinib showed a somewhat higher response rate (19%) but failed to improve OS compared to sorafenib [30], and its availability is limited as it is only approved in systemic first-line [4]. Given the lack of an established treatment with long-term survival benefit after first-line atezolizumab/bevacizumab and the poor prognosis of patients with advanced stage HCC, this patient population is considered appropriate to study novel therapeutic strategies.

In advanced melanoma patients, immune checkpoint inhibitors combined with FMT from immunotherapy responders to immunotherapy-refractory patients or from healthy donors to previously untreated patients demonstrated good tolerability and promising efficacy in early phase clinical trials [15–17]. Whether microbial composition has a predictive value for immune checkpoint inhibitor therapy remains under investigation, as is to studying algorithms that may predict response to immunotherapy [31,32]. Based on these emerging clinical data and on the fact that the gut microbiota is often altered in HCC and chronic liver diseases [10,11], we will conduct the FAB-HCC pilot study in order to evaluate the safety and efficacy of FMT combined with atezolizumab plus bevacizumab in HCC patients with failure of prior atezolizumab plus bevacizumab. Although the sample size is limited, the study aims to generate mechanistical hypotheses, which will help to define the potential of FMT as add-on intervention in the systemic treatment of advanced HCC, with the potential to improve efficacy of immunotherapy or even overcome resistance.

## Supporting information

S1 Table List of routine laboratory assessments. Abbreviations: HBV, hepatitis B virus; HBsAg, hepatitis B surface antigen; HBcAb, hepatitis B core antibody; HCV, hepatitis C virus; HIV, human immunodeficiency virus; DNA, deoxyribonucleic acid; RNA, ribonucleic acid.
(DOCX)

S1 File Clinical study protocol. (PDF)

S2 File SPIRIT 2013 Checklist. (PDF)

## Abbreviations

AESI: Adverse event of special interest
BOR: Best overall response
CTCAE: Common Terminology Criteria for Adverse Events
CR: Complete response
FMT: Fecal microbiota transplant
GMP: Good manufacturing practice
HCC: Hepatocellular carcinoma
ICI: Immune checkpoint inhibitor
IMP: Investigational Medicinal Product
IQR: Interquartile range
mRECIST: Modified Response Evaluation Criteria in Solid Tumors
MASH: Metabolic dysfunction-associated steatohepatitis
NCI: National cancer institute
OS: Overall survival
PBMCs: Peripheral blood mononuclear cells
PD: Progressive disease
PD-(L)1: Programmed cell death protein 1 (ligand) 1
PFS: Progression-free survival
PR: Partial response
Q6h: Every 6 hours
RECIST 1.1: Response Evaluation Criteria in Solid Tumors, Version 1.1
SD: Stable disease
TACE: Transarterial chemoembolization
VEGF: Vascular endothelial growth factor.

## References

[1] European Association for the Study of the Liver. EASL clinical practice guidelines: management of hepatocellular carcinoma. J Hepatol. 2018;69(1):182–236. doi: 10.1016/j.jhep.2018.03.019 29628281

[2] Pinter M, Jain RK, Duda DG. The current landscape of immune checkpoint blockade in hepatocellular carcinoma: a review. JAMA Oncol. 2021;7(1):113–23. doi: 10.1001/jamaoncol.2020.3381 33090190 PMC8265820

[3] Finn RS, Qin S, Ikeda M, Galle PR, Ducreux M, Kim T-Y, et al. Atezolizumab plus bevacizumab in unresectable hepatocellular carcinoma. N Engl J Med. 2020;382(20):1894–905. doi: 10.1056/NEJMoa1915745 32402160

[4] Bruix J, Chan S, Galle P, Rimassa L, Sangro B. Systemic treatment of hepatocellular carcinoma: An EASL position paper. J Hepatol. 2021;75(2):960–73. doi: insert_doi_here 34256065 10.1016/j.jhep.2021.07.004

[5] Pinter M, Scheiner B, Peck-Radosavljevic M. Immunotherapy for advanced hepatocellular carcinoma: a focus on special subgroups. Gut. 2021;70(1):204–14. doi: 10.1136/gutjnl-2020-321702 32747413 PMC7788203

[6] Abou-Alfa GK, Lau G, Kudo M, Chan SL, Kelley RK, Furuse J, et al. Tremelimumab plus Durvalumab in Unresectable Hepatocellular Carcinoma. NEJM Evid. 2022;1(8):EVIDoa2100070. doi: 10.1056/EVIDoa2100070 38319892

[7] Singal AG, Llovet JM, Yarchoan M, Mehta N, Heimbach JK, Dawson LA, et al. AASLD Practice Guidance on prevention, diagnosis, and treatment of hepatocellular carcinoma. Hepatology. 2023;78(6):1922–65. doi: 10.1097/HEP.0000000000000466 37199193 PMC10663390

[8] Ducreux M, Zhu AX, Cheng A-L, Galle PR, Ikeda M, Nicholas A, et al. IMbrave150: Exploratory analysis to examine the association between treatment response and overall survival (OS) in patients (pts) with unresectable hepatocellular carcinoma (HCC) treated with atezolizumab (atezo) + bevacizumab (bev) versus sorafenib (sor). JCO. 2021;39(15_suppl):4071–4071. doi: 10.1200/jco.2021.39.15_suppl.4071

[9] Pinter M, Scheiner B, Pinato DJ. Immune checkpoint inhibitors in hepatocellular carcinoma: emerging challenges in clinical practice. Lancet Gastroenterol Hepatol. 2023;8(8):760–70. doi: 10.1016/S2468-1253(23)00147-4 37327807

[10] Schwabe RF, Greten TF. Gut microbiome in HCC - Mechanisms, diagnosis and therapy. J Hepatol. 2020;72(2):230–8. doi: 10.1016/j.jhep.2019.08.016 31954488

[11] Behary J, Amorim N, Jiang X-T, Raposo A, Gong L, McGovern E, et al. Gut microbiota impact on the peripheral immune response in non-alcoholic fatty liver disease related hepatocellular carcinoma. Nat Commun. 2021;12(1):187. doi: 10.1038/s41467-020-20422-7 33420074 PMC7794332

[12] Routy B, Le Chatelier E, Derosa L, Duong CPM, Alou MT, Daillère R, et al. Gut microbiome influences efficacy of PD-1-based immunotherapy against epithelial tumors. Science. 2018;359(6371):91–7. doi: 10.1126/science.aan3706 29097494

[13] Matson V, Fessler J, Bao R, Chongsuwat T, Zha Y, Alegre M-L, et al. The commensal microbiome is associated with anti-PD-1 efficacy in metastatic melanoma patients. Science. 2018;359(6371):104–8. doi: 10.1126/science.aao3290 29302014 PMC6707353

[14] Gopalakrishnan V, Spencer CN, Nezi L, Reuben A, Andrews MC, Karpinets TV, et al. Gut microbiome modulates response to anti-PD-1 immunotherapy in melanoma patients. Science. 2018;359(6371):97–103. doi: 10.1126/science.aan4236 29097493 PMC5827966

[15] Baruch EN, Youngster I, Ben-Betzalel G, Ortenberg R, Lahat A, Katz L, et al. Fecal microbiota transplant promotes response in immunotherapy-refractory melanoma patients. Science. 2021;371(6529):602–9. doi: 10.1126/science.abb5920 33303685

[16] Davar D, Dzutsev AK, McCulloch JA, Rodrigues RR, Chauvin J-M, Morrison RM, et al. Fecal microbiota transplant overcomes resistance to anti-PD-1 therapy in melanoma patients. Science. 2021;371(6529):595–602. doi: 10.1126/science.abf3363 33542131 PMC8097968

[17] Routy B, Lenehan JG, Miller WH Jr, Jamal R, Messaoudene M, Daisley BA, et al. Fecal microbiota transplantation plus anti-PD-1 immunotherapy in advanced melanoma: a phase I trial. Nat Med. 2023;29(8):2121–32. doi: 10.1038/s41591-023-02453-x 37414899

[18] Lencioni R, Llovet JM. Modified RECIST (mRECIST) assessment for hepatocellular carcinoma. Semin Liver Dis. 2010;30(1):52–60. doi: 10.1055/s-0030-1247132 20175033 PMC12268942

[19] Finn RS, Qin S, Ikeda M, Galle PR, Ducreux M, Kim T-Y, et al. IMbrave150: Updated overall survival (OS) data from a global, randomized, open-label phase III study of atezolizumab (atezo) + bevacizumab (bev) versus sorafenib (sor) in patients (pts) with unresectable hepatocellular carcinoma (HCC). JCO. 2021;39(3_suppl):267–267. doi: 10.1200/jco.2021.39.3_suppl.267

[20] Agency E-E. Atezolizumab (Tecentriq) [summary of product characteristics]. 2021.

[21] Bruix J, Qin S, Merle P, Granito A, Huang Y-H, Bodoky G, et al. Regorafenib for patients with hepatocellular carcinoma who progressed on sorafenib treatment (RESORCE): a randomised, double-blind, placebo-controlled, phase 3 trial. Lancet. 2017;389(10064):56–66. doi: 10.1016/S0140-6736(16)32453-9 27932229

[22] Abou-Alfa GK, Meyer T, Cheng A-L, El-Khoueiry AB, Rimassa L, Ryoo B-Y, et al. Cabozantinib in patients with advanced and progressing hepatocellular carcinoma. N Engl J Med. 2018;379(1):54–63. doi: 10.1056/NEJMoa1717002 29972759 PMC7523244

[23] Zhu AX, Kang Y-K, Yen C-J, Finn RS, Galle PR, Llovet JM, et al. Ramucirumab after sorafenib in patients with advanced hepatocellular carcinoma and increased α-fetoprotein concentrations (REACH-2): a randomised, double-blind, placebo-controlled, phase 3 trial. Lancet Oncol. 2019;20(2):282–96. doi: 10.1016/S1470-2045(18)30937-9 30665869

[24] Kump PK, Krause R, Allerberger F, Högenauer C. Faecal microbiota transplantation--the Austrian approach. Clin Microbiol Infect. 2014;20(11):1106–11. doi: 10.1111/1469-0691.12801 25274251

[25] Cammarota G, Ianiro G, Tilg H, Rajilić-Stojanović M, Kump P, Satokari R, et al. European consensus conference on faecal microbiota transplantation in clinical practice. Gut. 2017;66(4):569–80. doi: 10.1136/gutjnl-2016-313017 28087657 PMC5529972

[26] Paramsothy S, Kamm MA, Kaakoush NO, Walsh AJ, van den Bogaerde J, Samuel D, et al. Multidonor intensive faecal microbiota transplantation for active ulcerative colitis: a randomised placebo-controlled trial. Lancet. 2017;389(10075):1218–28. doi: 10.1016/S0140-6736(17)30182-4 28214091

[27] Costello SP, Hughes PA, Waters O, Bryant RV, Vincent AD, Blatchford P, et al. Effect of fecal microbiota transplantation on 8-week remission in patients with ulcerative colitis: a randomized clinical trial. JAMA. 2019;321(2):156–64. doi: 10.1001/jama.2018.20046 30644982 PMC6439766

[28] Ni J, Shen TD, Chen EZ, Bittinger K, Bailey A, Roggiani M, et al. A role for bacterial urease in gut dysbiosis and Crohn’s disease. Sci Transl Med. 2017;9(416). 10.1126/scitranslmed.aah6888 PMC5808452 29141885

[29] Cheng A-L, Kang Y-K, Chen Z, Tsao C-J, Qin S, Kim JS, et al. Efficacy and safety of sorafenib in patients in the Asia-Pacific region with advanced hepatocellular carcinoma: a phase III randomised, double-blind, placebo-controlled trial. Lancet Oncol. 2009;10(1):25–34. doi: 10.1016/S1470-2045(08)70285-7 19095497

[30] Yamashita T, Kudo M, Ikeda K, Izumi N, Tateishi R, Ikeda M, et al. REFLECT-a phase 3 trial comparing efficacy and safety of lenvatinib to sorafenib for the treatment of unresectable hepatocellular carcinoma: an analysis of Japanese subset. J Gastroenterol. 2020;55(1):113–22. doi: 10.1007/s00535-019-01642-1 31720835 PMC6942573

[31] Robinson I, Hochmair MJ, Schmidinger M, Absenger G, Pichler M, Nguyen VA, et al. Assessing the performance of a novel stool-based microbiome test that predicts response to first line immune checkpoint inhibitors in multiple cancer types. Cancers (Basel). 2023;15(13):3268. doi: 10.3390/cancers15133268 37444378 PMC10339964

[32] Gharaibeh RZ, Jobin C. Microbiota and cancer immunotherapy: in search of microbial signals. Gut. 2019;68(3):385–8. doi: 10.1136/gutjnl-2018-317220 30530851 PMC6580757

