# Supplementary material for: Study protocol: Fecal Microbiota Transplant combined with Atezolizumab/Bevacizumab in Patients with Hepatocellular Carcinoma who failed to achieve or maintain objective response to Atezolizumab/Bevacizumab – the FAB-HCC pilot study
Source: PLoS One. 2025 Apr 15;20(4):e0321189. doi: 10.1371/journal.pone.0321189 (PMC11999108; doi:10.1371/journal.pone.0321189)
Supplement: S1 Table — Abbreviations: HBV, hepatitis B virus; HBsAg, hepatitis B surface antigen; HBcAb, hepatitis B core antibody; HCV, hepatitis C virus; HIV, human immunodeficiency virus; DNA, deoxyribonucleic acid; RNA, ribonucleic acid. (DOCX) [file pone.0321189.s001.docx]

| **Supplemental Table 1**. List of routine laboratory assessments. | |
| --- | --- |
| **Panel** | **Parameters** |
| **Hematology** | White blood cell count (incl. differential count, i.e., neutrophils, eosinophils, basophils, monocytes, lymphocytes, other cells), red blood cell count, hemoglobin, hematocrit, platelet count |
| **Chemistry** | Sodium, potassium, chloride, phosphate, calcium, glucose, blood urea nitrogen or urea, creatinine, total protein, albumin, total bilirubin, alkaline phosphatase, alanine-aminotransferase, aspartate-aminotransferase, gamma glutamyl transferase, lactate dehydrogenase, C-reactive protein |
| **Coagulation** | Thromboplastin time (Owen), international normalized ratio, and activated partial thromboplastin time |
| **Hormones** | Thyroid stimulating hormone, free triiodothyronine, free thyroxine |
| **Tumor marker** | Alpha-fetoprotein |
| **Virology** | HIV serology, HBV serology (HBsAg, HBcAb, HBV DNA), HCV serology (HCV antibody, and if positive then HCV RNA) |
| **Urine analysis** | Dipstick (pH, blood, leukocytes, protein, ketones, glucose) |
| **Pregnancy test** | All women of childbearing potential will have a serum pregnancy test within 14 days before Day 1 of Cycle 1: Urine pregnancy tests will be performed at specified visits. If a urine pregnancy test is positive, it must be confirmed by a serum pregnancy test. |
| **Abbreviations:** HBV, hepatitis B virus; HBsAg, hepatitis B surface antigen; HBcAb, hepatitis B core antibody; HCV, hepatitis C virus; HIV, human immunodeficiency virus; DNA, deoxyribonucleic acid; RNA, ribonucleic acid; | |
