# Supplementary material for: Study protocol: Fecal Microbiota Transplant combined with Atezolizumab/Bevacizumab in Patients with Hepatocellular Carcinoma who failed to achieve or maintain objective response to Atezolizumab/Bevacizumab – the FAB-HCC pilot study
Source: PLoS One. 2025 Apr 15;20(4):e0321189. doi: 10.1371/journal.pone.0321189 (PMC11999108; doi:10.1371/journal.pone.0321189)
Supplement: S1 File — (PDF) [file pone.0321189.s002.pdf]

Clinical Study Protocol

Fecal Microbiota Transplant (FMT) combined with Atezolizumab plus Bevacizumab in Patients with HepatoCellular Carcinoma who failed to respond to prior Immunotherapy – the FAB-HCC pilot study

FMT in IT-refractory HCC – FAB-HCC pilot study

PROTOCOL NUMBER: FAB0001

VERSION NUMBER: 7

DATE: 02.11.2023

EUDRACT NUMBER: 2022-000234-42

**Test drug (IMP) and Pharmaceutical Company**	Atezolizumab plus Bevacizumab in combination with fecal microbiota transplantation (FMT)<br><br>Roche
**Protocol author**	Ap.Prof. Priv.-Doz. Dr. Matthias Pinter, PhD<br><br>Dr. Katharina Pomej
**Principal investigator**	Ap.Prof. Priv.-Doz. Dr. Matthias Pinter, PhD
**Document type**	Clinical study protocol
**Study phase**	Phase 2a (pilot study)
**Date**	02.11.2023
**Number of pages**	41

1 SPONSOR, INVESTIGATOR, MONITOR AND SIGNATURES

Sponsor/or representative (AMG §§ 2a, 31, 32)

Univ.-Prof. Dr. Michael Trauner

Department for Internal Medicine III, Division of Gastroenterology and Hepatology

Medical University of Vienna, Austria

Signature	Date
______________________	______________________

Investigator (AMG §§ 2a, 35, 36)

Ap.Prof. Priv.-Doz. Dr. Matthias Pinter, PhD

Department for Internal Medicine III, Division of Gastroenterology and Hepatology

Medical University of Vienna, Austria

Signature	Date
______________________	______________________

Monitor/ or Representative of CRO (AMG §§ 2a, 33, 34)

Anita Vamosi, MSc

KKS – Clinical Trials Coordination Center

Medical University of Vienna

Signature	Date
______________________	______________________

Statistician

Univ. Prof. Dr. Harald Heinzl

Centre for Medical Statistics, Informatics and Intelligent Systems, Institute of Medical Biometrics

Medical University of Vienna

Signature	Date
______________________	______________________

Clinical Trial Center:

Department for Internal Medicine III, Division of Gastroenterology and Hepatology

Medical University of Vienna, Austria

2 PROTOCOL SYNOPSIS

TITLE	**Fecal Microbiota Transplant (FMT) combined with Atezolizumab plus Bevacizumab in Patients with HepatoCellular Carcinoma who failed to respond to prior Immunotherapy – the FAB-HCC pilot study**
OBJECTIVES	**Primary Objective**<br>• *Safety as measured by incidence and severity of treatment-related adverse events, with severity determined according to National Cancer Institute (NCI) Common Terminology Criteria for Adverse Events (CTCAE) version 5.0*<br>**Secondary Objectives**<br>• *Efficacy as assessed by best radiological response according to mRECIST and RECIST v1.1 criteria*<br>• *Efficacy as assessed by objective response rate and disease control rate*<br>• *Efficacy as assessed by progression-free survival and overall survival*<br>• *Quality of life assessed by EQ-5D-5L questionnaire*
DESIGN / PHASE	*Open phase IIa pilot study*
STUDY PLANNED DURATION	**First patient First visit**: *Q2/2022*<br>**Last patient First visit**: *Q1/2024*<br>**Last patient Last visit**: *Q2/2024*
CENTER(S) / COUNTRY(IES)	*Medical University of Vienna*<br>*Austria, Europe*
PATIENTS / GROUPS	*12 Patients with HCC who failed to achieve or maintain a complete or partial response (according to mRECIST) to atezolizumab plus bevacizumab*<br>*No Randomization, no stratification*
INCLUSION CRITERIA	• *See Section 7.1.2 for detailed inclusion criteria*
EXCLUSION CRITERIA	• *See Section 7.1.3 for detailed exclusion criteria*
INVESTIGATIONAL DRUG	***MED 1: Atezolizumab** will be administered by IV infusion at a fixed dose of **1200mg** on Day 1 of each 21-day cycle*<br>***MED 2: Bevacizumab** will be administered by IV infusion at a dose of **15mg/kg** on Day 1 of each 21-day cycle*
CONCOMITANT MEDICATION	*Any medication (e.g.: prescription drugs, over-the-counter drugs, vaccines, herbal or homeopathic remedies, nutritional supplements) should be reported to the investigator and recorded in the concomitant medications eCRF.*<br>***Not allowed**: other anti-cancer treatments, live attenuated vaccines, systemic immunostimulatory agents (IFNs, IL-2)*

EXPLORATORY ENDPOINTS	• *Effect of FMT on recipient gut microbiota composition, diversity (alpha and beta), rate of change from baseline and similarity to donor stool composition over time as well as comparison of responders and non-responders.*<br>• *Effect of FMT on immune activity in the gut*<br>• *Metagenome assemblies and functional profiling using shotgun metagenomic analysis of donor and recipient stool samples before and after FMT*<br>• *Single cell analyses of circulating immune cells before and after FMT*<br>• *Serum and stool metabolomic and lipidomic signatures before and after FMT*
STATISTICAL METHODOLOGY	**Primary Endpoint**<br>*Safety as measured by incidence and severity of treatment-related adverse events, with severity determined according to National Cancer Institute (NCI) Common Terminology Criteria for Adverse Events (CTCAE) version 5.0*<br>**Research hypothesis:**<br>*Fecal microbiota transplantation in patients with advanced hepatocellular carcinoma receiving immunotherapy is safe and feasible*<br>**Sample size calculation:**<br>*As this study represents a pilot study evaluating safety and feasibility as well as due to the one-arm design of this trial no formal sample size calculation was performed.*<br>**Statistical methodology:**<br>*Primary objective: Descriptive statistics*<br>*Secondary objectives: Descriptive statistics. Progression-free survival will be defined as the time from FMT to the date of radiological disease progression or death. Overall survival will be defined as the time from FMT to the date of death. Patients who are still alive at the end of the study will be censored with their last follow-up date.*<br>*Exploratory endpoints: Statistical methods for the exploratory endpoints will be described in the respective sections.*<br>**Interim analysis:**<br>*Interim analysis after six subjects underwent at least one radiological follow-up: If disease control cannot be achieved in ≥50% (3 subjects) of the first 6 subjects, the study will be terminated prematurely.*

3 LIST OF ABBREVIATIONS

ADR	Adverse Drug Reaction
AE	Adverse Event
AFP	α-fetoprotein
ALT	Alanine Aminotransferase
AMG	Arzneimittelgesetz
AST	Aspartate Aminotransferase
CRF	Case Report Form
CRO	Clinical Research Organization
CSR	Clinical Study Report
CT	Computed Tomography
DCR	Disease control rate
DOH	Declaration of Helsinki
EASL	European Association for the Study of the Liver
EC	Ethics Committee
ECG	Electrocardiography
ECOG	Eastern Cooperative Oncology Form
eCRF	Electronic case report form
EHS	Extrahepatic spread
EOS	End of Study
FMT	Fecal microbiota transplant
GCP	Good Clinical Practice
GGT	Gamma-glutamyltransferase
GI	Gastrointestinal
HBsAg	Hepatitis B Surface Antigen
HBV	Hepatitis B Virus
HCC	Hepatocellular carcinoma
HCV	Hepatitis C Virus
HIV	Human Immunodeficiency Virus
ICH	International Conference on Harmonization
IEC	Independent Ethics Committee
IMP	Investigational Medicinal Product
ISF	Investigator Site File
ISO	International Standardisation Organization

IRB	Institutional Review Board
IRR	Infusion-related reaction
ICI	Immune checkpoint inhibitor
IRR	Infusion-related reaction
ITT	Intention-to-treat (population)
KKS	Koordinationszentrum für Klinische Studien
Mo	Months
mRECIST	Modified Response Evaluation Criteria in Solid Tumors
MRI	Magnetic resonance imaging
MWA	Microwave ablation
NASH	Non-alcoholic steatohepatitis
NCI	National cancer institute
NE	Not evaluable
ORR	Overall response rate
OS	Overall survival
PD	Progressive disease
PD-1	Programmed cell death protein 1
PD-L1	Programmed cell death 1 ligand 1
PFS	Progression-free survival
PR	Partial response
Q3W	Every 3 weeks
RECIST 1.1	Response Evaluation Criteria in Solid Tumors, Version 1.1
RFA	Radiofrequency ablation
RBC	Red Blood Count
SAE	Serious Adverse Event
SAP	Statistical analysis plan
SAR	Serious Adverse Reaction
SD	Stable disease
SOP	Standard Operating Procedure
SUSAR	Suspected Unexpected Serious Adverse Reaction
TACE	Transarterial chemoembolization
TKI	Tyrosine kinase inhibitor
TMF	Trial Master File
VEGF	Vascular endothelial growth factor
WBC	White Blood Cells
WHO	World Health Organization

4 TABLE OF CONTENTS

1 SPONSOR, INVESTIGATOR, MONITOR AND SIGNATURES 3
2 PROTOCOL SYNOPSIS 4
3 LIST OF ABBREVIATIONS 6
4 TABLE OF CONTENTS 8
5 BACKGROUND INFORMATION 12
5.1 Background 12
5.2 Study rationale and benefit risk assessment 14
6 STUDY OBJECTIVES (HYPOTHESIS) 14
6.1 Primary objective (Hypothesis) 14
6.2 Secondary objectives (Hypothesis) 15
6.3 Exploratory objectives 15
7 STUDY DESIGN 15
7.1 Study population 15
7.1.1 Subject population 15
7.1.2 Inclusion criteria 15
7.1.3 Exclusion criteria 16
7.1.4 Females of childbearing potential 16
7.1.5 Study duration 16
7.1.6 Withdrawal and replacement of subjects 17
7.1.7 Premature termination of the study 17
8 METHODOLOGY 17
8.1 Study medication 18
8.1.1 Dosage and administration of Atezolizumab 18
8.1.2 Dosage and administration of Bevacizumab 19
8.1.3 Study drug interruption or discontinuation 20
8.1.4 Study drug interruption 20
8.1.5 Study drug premature permanent discontinuation 20
8.1.6 Study-drug delivery & drug storage conditions 20
8.1.7 IMP administration & handling 20
8.1.8 Drug accountability 21
8.1.9 Procedures to assess subjects' compliance 21
8.1.10 Concomitant medication 21
8.2 Study procedures 21
8.2.1 General rules for trial procedures 21
8.2.2 Screening investigation 21
8.2.3 Study assessments 22
8.2.4 Laboratory assessments 22
8.2.5 Fecal microbiota transplant (FMT) 22

8.2.6 Imaging assessments 25
8.2.7 Patient-reported outcomes instruments 25
8.2.8 End-of-study (EOS) examination 25
8.2.9 Definition of the end of the trial 25
9 SAFETY DEFINITIONS AND REPORTING REQUIREMENTS 25
9.1 Averse events (AEs) 25
9.1.1 Summary of known and potential risks associated with Atezolizumab 25
9.1.2 Summary of known and potential risks associated with Bevacizumab 26
9.1.3 Definition of adverse events 26
9.1.4 Adverse event reporting period 27
9.2 Serious adverse events (SAEs) 27
9.2.1 Hospitalization – Prolongation of existing hospitalization 27
9.2.2 SAEs related to investigational drug 27
9.3 Suspected unexpected serious adverse reactions (SUSARs) 27
9.4 Adverse events of special interest (AESI; immediately reportable to the sponsor) 28
9.5 Severity of adverse events 28
9.6 Relationship to study drug 29
9.7 Reporting procedures 30
9.7.1 Reporting procedures for SAEs 30
9.7.2 Reporting procedures for SUSAR 30
9.7.3 Reporting procedures for AESIs 31
9.7.4 Reporting procedures for pregnancies 31
9.7.4.1 Pregnancies in female patients 31
9.7.4.2 Pregnancies in female partners of male patients 31
10 FOLLOW-UP 31
10.1 Follow-up of study participants including follow-up of adverse events 31
10.2 Treatment after end of study 32
11 STATISTICAL METHODOLOGY AND ANALYSIS 32
11.1 Analysis sets 32
11.2 Sample size considerations 32
11.3 Relevant protocol deviations 32
11.4 Endpoint analysis 33
11.4.1 Primary endpoint analysis 33
11.4.2 Secondary endpoint analysis 33
11.4.3 Exploratory objectives 33
11.5 Interim analysis 35
11.6 Software program(s) 35
12 DOCUMENTATION AND DATA MANAGEMENT 35
12.1 Documentation of study results 35
12.1.1 Case report form (CRF) 35

12.1.2 Data collection 35
12.2 Safekeeping 35
12.3 Quality control and quality assurance 36
12.3.1 Periodic Monitoring 36
12.3.2 Audit and inspections 36
12.4 Reporting and publication 36
12.4.1 Publication of study results 36
13 ETHICAL AND LEGAL ASPECTS 36
13.1 Informed consent of subjects 36
13.2 Acknowledgement / approval of the study 37
13.2.1 Changes in the conduct of the study 37
13.3 Insurance 37
13.4 Confidentiality 37
13.5 Ethics and good clinical practice (GCP) 38
14 REFERENCES 39
15 TABLES AND FIGURES 41

PERIODS	Name	SCREENING		TREATMENT									FOLLOW-UP
	Duration	28 days											28 days
VISITS	Number	1	2	3	4	5	6	7	8	9			
	Name	Screening	FMT	Cycle 1	Cycle 2	Cycle 3	Cycle 4	Cycle 5	Cycle 6	Cycle 7	Further Cycles	End of study	Follow-up
	Time	Days -28 to -1	Day 0	Day 1 (± 3 days)	Week 3 (± 5 days)	Week 6 (± 5 days)	Week 9 (± 5 days)	Week 12 (± 5 days)	Week 15 (± 5 days)	Week 18 (± 5 days)		(± 5 days)	Day 28 (± 5 days)
Informed Consent		X											
Inclusion / Exclusion Criteria		X											
Medical History		X		X	X	X	X	X	X	X	X	X	X
ECOG Performance Status		X		X	X	X	X	X	X	X	X	X	X
Concomitant/change in medication		X		X	X	X	X	X	X	X	X	X	X
Physical Examination		X		X	X	X	X	X	X	X	X	X	X
Body weight and height		X		X	X	X	X	X	X	X	X	X	X
Vital Signs (BP, PR, Temperature)		X		X	X	X	X	X	X	X	X	X	X
12-lead ECG[a]		X											
Laboratory Tests		X		X	X	X	X	X	X	X	X	X	X
Blood Sampling		X		X	X	X	X	X		X			
Virological Tests		X											
Urine Analysis		X		X	X	X	X	X	X	X	X	X	
Stool Sampling		X		X	X[b]	X	X	X		X			
Pregnancy Test[c]		X											
Colonoscopy + FMT			X										
Imaging Assessments		X				X				X	X[d]		
Quality of Life Assessments		X		X		X		X		X	X[e]	X	X
Study Drug Administration				X	X	X	X	X	X	X	X		
Adverse Events		X		X	X	X	X	X	X	X	X	X	X

Table 1 Visit and assessment schedule.

[a]12-lead ECG during screening and on Day 1. Further ECGs should be performed upon clinical suspicion.
[b]At week 1, week 2, and week 3.
[c]Repetitive pregnancy assessment in women with childbearing potential.
[d]Every 4th cycle.
[e]Every other visit (odd cycles).

5 BACKGROUND INFORMATION

5.1 Background

Hepatocellular carcinoma (HCC) represents 90% of all primary liver cancers and the second most common cause of cancer-related death [1]. Resection, local ablation, and liver transplantation are potential curative therapies, but are reserved for early stage HCC. Unfortunately, most patients are diagnosed at an advanced tumor stage, where only palliative treatment options are available. Patients with liver-limited, multifocal HCC are usually treated with transarterial chemoembolization (TACE), while those with macrovascular tumor invasion or extrahepatic metastases are typical candidates for systemic therapy [1]. Until recently, the tyrosine kinase inhibitors (TKIs) sorafenib and lenvatinib were indicated in systemic first-line, and the TKIs regorafenib and cabozantinib, as well as the monoclonal antibody against vascular endothelial growth factor (VEGF) receptor-2, ramucirumab, have been approved for sorafenib-pretreated patients [2].

Encouraging data on immune checkpoint inhibitors (ICIs) in HCC from phase II trials directed the research progressively towards immunotherapy. However, subsequent randomized controlled phase III trials testing programmed cell death protein 1 (PD-1)-targeted monotherapy with nivolumab or pembrolizumab failed to significantly improve survival endpoints [3]. These setbacks suggested that combined systemic approaches may be needed to implement immunotherapy in HCC. VEGF's promotion of immunosuppression in the tumor microenvironment was the rationale to combine ICIs with VEGF-targeted agents [3]. In a pivotal phase III trial, the combination of atezolizumab (monoclonal antibody against programmed cell death 1 ligand 1 (PD-L1)) plus bevacizumab (monoclonal antibody against VEGF) improved both primary endpoints overall survival (OS) and progression-free survival versus sorafenib, and demonstrated good safety and improved quality of life data (Table 2) [4]. Consequently, atezolizumab plus bevacizumab was implemented as the new standard of care in systemic front-line treatment of HCC for the majority of patients [2,5].

	OS, median	OS, HR (95%CI)	PFS, median	PFS, HR (95%CI)	ORR (RECISTv1.1)	DCR (RECISTv1.1)
**First interim analysis**						
Atezolizumab + bevacizumab	NE	0.58 (0.42-0.79)	6.8 mo	0.59 (0.47-0.76)	27%	74%
Sorafenib	13.2 mo		4.3 mo		12%	55%
**Updated follow-up**						
Atezolizumab + bevacizumab	19.2 mo	0.66 (0.52-0.85)	6.9 mo	0.65 (0.53-0.81)	30%	74%
Sorafenib	13.4 mo		4.3 mo		11%	55%

Abbreviations: DCR, disease control rate; mo, months; NE, not evaluable; ORR, objective response rate; overall OS, overall survival; PFS, progression-free survival; RECISTv1.1, Response Evaluation Criteria in Solid Tumors version 1.1

Table 2 Main efficacy results from the global, randomized, open-label phase III study (IMbrave 150) of atezolizumab plus bevacizumab versus sorafenib in patients with unresectable hepatocellular carcinoma.

Even though these results represent a milestone in the systemic treatment of HCC, only around one-third of patients responds to atezolizumab/bevacizumab (Table 2) [4]. While patients with stable disease (SD) still show improved overall survival compared to subjects with progressive disease (PD), patients with complete (CR) or partial response (PR) are those most likely to derive a long-term survival benefit from immunotherapy (Figure 1) [6]. Currently, there is no established biomarker to predict response to immunotherapy [3].

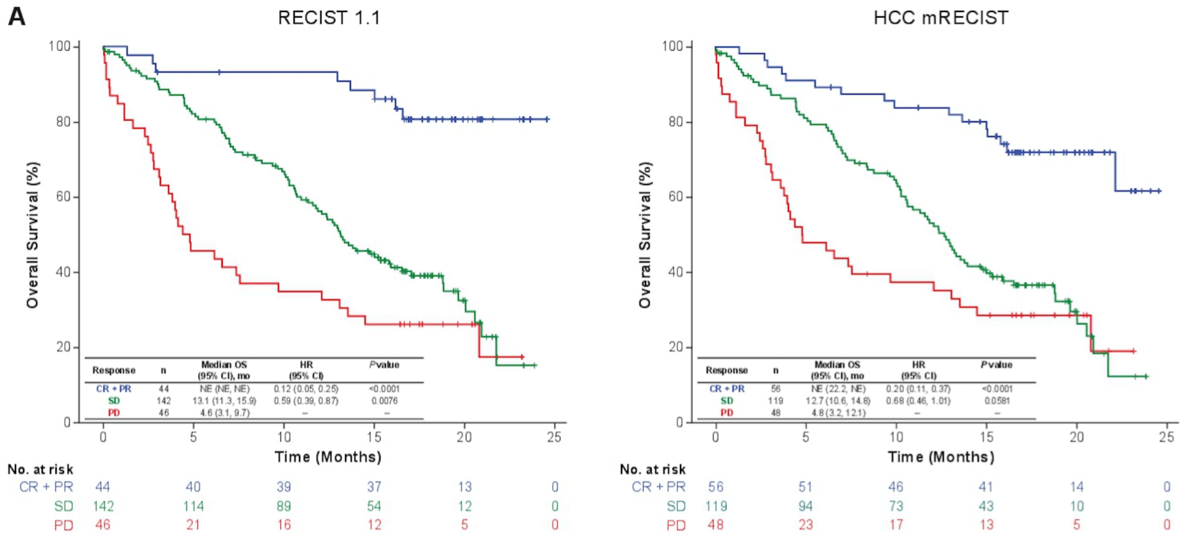

Figure 1 Overall survival in patients treated with atezolizumab plus bevacizumab achieving confirmed complete (CR)/partial response (PR) vs. stable disease (SD) vs. progressive disease (PD) using 4-month landmarks (from Ducreux M et al. Abstract # 4071, presented at ASCO Annual Meeting 2021).

The gut microbiota is often altered in chronic liver diseases and HCC, and may modulate cancer-promoting and cancer-suppressing pathways associated with immunity and inflammation [7]. In non-alcoholic steatohepatitis (NASH)-related HCC, gut dysbiosis promotes peripheral immunosuppression [8]. Increasing evidence suggests that the gut microbiome may influence response to cancer immunotherapy [9-11]. Hence, strategies to modulate the gut microbiome (i.e., fecal microbiota transplant (FMT)) may help to improve efficacy of ICIs or even overcome resistance to immunotherapy. Indeed, in preclinical studies, FMT obtained from patients responding to ICIs into mice (germ-free or antibiotic-treated) enhanced the efficacy of anti-PD-(L)1 treatment and augmented T cell response, while FMT from non-responders did not [9-11]. Two recently published clinical pilot studies confirmed the feasibility and safety of this innovative approach in humans [12,13]. In a phase I clinical trial, 10 metastatic melanoma patients with confirmed progression on PD-1-targeted immunotherapy received FMT from donors with metastatic melanoma who had achieved complete remission for at least 12 months with PD-1-targeted immunotherapy. Reintroduction of anti-PD-1 treatment after FMT led to 2 partial responses and 1 complete response. The safety profile was excellent with mild bloating being the only FMT-related adverse event [12]. The second clinical trial investigated the safety and efficacy of responder-derived FMT in combination with PD-1-targeted immunotherapy in patients with melanoma primary refractory to anti-PD-1 treatment. Of 15 patients evaluable for radiological response, 3 achieved complete (n=1) or partial (n=2) remission and 3 patients had durable stable disease for more than 12 months. No relevant FMT-related adverse events were reported [13].

5.2 Study rationale and benefit risk assessment

The combination of atezolizumab plus bevacizumab represents the new reference standard in systemic front-line therapy for advanced stage HCC [2,4]. Patient who respond to this treatment can derive a long-term survival benefit [6]. However, only around one-third of patients treated with atezolizumab/bevacizumab achieves CR or PR, while around 20% have a primary resistance reflected by disease progression already at the first radiological evaluation. Patients who achieve stable disease (~40%) have an initial clinical benefit, but most of them will eventually show progression of the disease as well [4].

Disease progression usually triggers a change in systemic treatment, but given that all available second-line agents have been tested in sorafenib-pretreated patients, no established second-line option exists after atezolizumab/bevacizumab. Thus, approved targeted therapies (i.e., sorafenib, lenvatinib, regorafenib, cabozantinib, ramucirumab) are recommended as per off-label availability [2]. However, these agents have limited efficacy (median OS improvement: <3 months vs. placebo; response rate: 2%-11%). Only lenvatinib showed a somewhat higher response rate (19%) but failed to improve OS compared to sorafenib, and its availability is limited as it is only approved in systemic first-line [2]. Given the lack of an established treatment with long-term survival benefit after first-line atezolizumab/bevacizumab and the poor prognosis of patients with advanced stage HCC, this patient population is considered appropriate to study novel therapeutic strategies.

In this pilot study, we will perform FMT from HCC patients who responded to PD-(L)1-based immunotherapy or from healthy donors to patients who progressed on atezolizumab/bevacizumab aiming to turn them into responders. We will also offer FMT to patients having achieved stable disease as best radiological response according to mRECIST[14] after the first 12 months of treatment, as it is unlikely that a response to atezolizumab/bevacizumab will occur after that time (median time to first response (range), 2.8 (1.2-7.2) months) [15].

FMT is an established treatment for patients with recurrent Clostridium difficile infection not responding to antibiotics, and has demonstrated some promising preliminary results in other gastrointestinal disorders as well as diseases outside the gastrointestinal tract [16,17]. FMT will be performed according to international recommendations [17].

Given that most patients with HCC suffer from concomitant liver cirrhosis, it is very likely that potential donors will have an underlying liver disease (e.g., chronic hepatitis B or C, alcoholic liver disease, non-alcoholic fatty liver disease)/liver cirrhosis. To ensure the safety of FMT recipients, patients with active viral hepatitis (positive PCR test) as underlying liver disease will be excluded from becoming donors. Additionally, stool from donors with liver cirrhosis can only be used for FMT in recipients having liver cirrhosis as well. Finally, the feasibility and safety of FMT from cancer patients responding to immunotherapy to cancer patients refractory to immunotherapy has already been demonstrated in clinical trials [12,13].

6 STUDY OBJECTIVES (HYPOTHESIS)

This pilot study will evaluate the safety, feasibility, and efficacy of FMT from patients with HCC who responded to PD-(L)1-based immunotherapy or from healthy donors to patients with HCC who failed to achieve or maintain a response to atezolizumab/bevacizumab.

6.1 Primary objective (Hypothesis)

Safety as measured by incidence and severity of treatment-related adverse events, with severity determined according to National Cancer Institute (NCI) Common Terminology Criteria for Adverse Events (CTCAE) version 5.0.

6.2 Secondary objectives (Hypothesis)

- Efficacy as assessed by best radiological response according to mRECIST and RECIST v1.1 criteria
- Efficacy as assessed by objective response rate and disease control rate
- Efficacy as assessed by progression-free survival and overall survival
- Quality of life as assessed EQ-5D-5L questionnaire

6.3 Exploratory objectives

- Effect of FMT on recipient gut microbiota composition, diversity (alpha and beta), rate of change from baseline and similarity to donor stool composition over time as well as comparison of responders and non-responders.
- Effect of FMT on immune activity in the gut
- Metagenome assemblies and functional profiling using shotgun metagenomic analysis of donor and recipient stool samples before and after FMT
- Single cell analyses of circulating immune cells before and after FMT
- Serum and stool metabolomic and lipidomic signatures before and after FMT

7 STUDY DESIGN

This is a single-center, pilot study (phase IIa) designed to evaluate the safety, feasibility, and efficacy of FMT from anti-PD-(L)1 responders or healthy donors to patients with HCC who failed to achieve or maintain a response to atezolizumab/bevacizumab.

7.1 Study population

7.1.1 Subject population

The study is planned to enroll a total of 12 patients with HCC who failed to achieve or maintain a complete or partial radiological response (according to mRECIST) to atezolizumab plus bevacizumab. The study aims to include mainly patients who progressed (according to mRECIST) on this treatment, but study participation will also be offered to patients who have achieved stable disease as best radiological response according to mRECIST after the first 12 months of treatment initiation, as it is unlikely that a response to atezolizumab/bevacizumab will occur after that time.

7.1.2 Inclusion criteria

Patients must meet the following criteria:

- Signed informed consent form
- Age ≥ 18 years
- Histologically or radiologically confirmed HCC
- Patients with progressive disease (according to mRECIST) during treatment with atezolizumab/bevacizumab (without or with prior complete or partial response as best radiological response according to mRECIST) OR patients with stable disease as best radiological response (according to mRECIST) after the first 12 months of atezolizumab/bevacizumab treatment
- Negative HIV test
- Patients with chronic hepatitis B must be under antiviral treatment and hepatitis B DNA must be <500 IU/mL
- Variceal status must be known and if present, adequate medical or endoscopic treatment is required
- ECOG Performance Status 0-1
- Child-Pugh class A-B8
- Adequate hematological and end-organ function, defined as follows:
    - AST and ALT < 10 x ULN

- Serum bilirubin <3.5 mg/dL
- Albumin ≥28 g/L
- Serum creatinine ≤ 1.5 mg/dL
- Hemoglobin ≥ 8 mg/dL
- Platelet count ≥ 50 G/L
- Leukocytes ≥ 2.5 G/L
- Patients not receiving therapeutic anticoagulation: INR ≤ 2.3 or thromboplastin time ≥ 40%

▪ Women of childbearing potential must agree to remain abstinent (refrain from heterosexual intercourse) or use contraceptive methods
▪ Men must agree to remain abstinent (refrain from heterosexual intercourse) or use a condom

7.1.3 Exclusion criteria

Patients with any of the following criteria are NOT allowed to participate:

▪ Known fibrolamellar carcinoma or mixed cholangiocellular carcinoma
▪ Massive tumor progression (>100% increase in target lesions or progression associated with significant clinical deterioration)
▪ Uncontrolled ascites
▪ Overt hepatic encephalopathy or concomitant treatment with rifaximin
▪ Prior allogeneic stem cell or solid organ transplantation
▪ Active or history of severe autoimmune disease
▪ History of idiopathic pulmonary fibrosis, organizing pneumonia (e.g., bronchiolitis obliterans), drug-induced pneumonitis, or idiopathic pneumonitis, or evidence of active pneumonitis
▪ Significant cardiovascular disease (such as New York Heart Association Class II or greater cardiac disease, myocardial infarction, or cerebrovascular accident) within 3 months prior to study inclusion or unstable angina
▪ Severe infection within 4 weeks prior to study inclusion
▪ Pregnant or breastfeeding women
▪ Treatment with systemic immunosuppressive medication with the following exceptions:
  - Acute, low-dose systemic immunosuppressant medication or a one-time pulse dose of systemic immunosuppressant medication (e.g., 48 hours of corticosteroids for contrast allergy)
  - Mineralocorticoids (e.g., fludrocortisone), corticosteroids for chronic obstructive pulmonary disease or asthma, or low-dose corticosteroids for adrenal insufficiency
▪ Significant vascular disease (e.g., peripheral arterial thrombosis) within 6 months prior to study inclusion
▪ Major surgery within 4 weeks prior to study inclusion or minor surgery (excluding placement of a vascular access device) within 3 days prior to study inclusion
▪ History of gastrointestinal fistula or perforation, or intraabdominal abscess within 6 months prior to study inclusion
▪ Serious, non-healing wound or active ulcer

7.1.4 Females of childbearing potential

Women of childbearing potential must agree to remain abstinent (refrain from heterosexual intercourse) or use contraceptive methods. Moreover, a pregnancy test, including analysis of Human Chorionic Gonadotrophin [HCG] in urine and serum sample, will be performed.

7.1.5 Study duration

The start of the study is defined as the date of the first patient's first visit. The end of the study is defined as the date when every patient enrolled had a follow-up of at least 20 weeks (2 scheduled follow-up imaging visits), if not progressed or deceased earlier.

7.1.6 Withdrawal and replacement of subjects

Criteria for withdrawal

Subjects may prematurely discontinue from the study at any time. Premature discontinuation from the study means that the subject did not undergo an end of study examination as planned per protocol.

Subjects must be withdrawn under the following circumstances:

- at their own request
- if the Investigator feels it would not be in the best interest of the subject to continue
- if the subject violates conditions laid out in the consent form / information sheet or disregards instructions by the study personal

In all cases, the reason why subjects are withdrawn must be recorded in detail in the CRF and in the subject's medical records. Should the study be discontinued prematurely, all study materials (completed, partially completed and empty CRFs) will be retained.

Follow-up of patients withdrawn from the study

In case of premature discontinuation after study drug intake, the investigations scheduled for the EOS visit will be performed at release from the hospital and at routine outpatient visit thereafter. The subjects will be advised that participation in these investigations is voluntary. Furthermore, they may request that from the time point of withdrawal no more data will be recorded and that all biological samples collected in the course of the study will be destroyed.

Replacement policy

Withdrawn (unacceptable toxicity) and drop-out patients may be replaced by the next free subject and will not be included in the sample size. Data of these patients will be considered in the intent to treat analysis.

7.1.7 Premature termination of the study

Based on previous phase III second-line studies in HCC [18-20], a disease control rate of less than 50% is below what would be expected. Thus, to ensure that a likely unsuccessful therapeutic strategy is not pursued, the study will stop enrollment prematurely if the disease control rate of the first 6 patients included is less than 50%.

Furthermore, the sponsor has the right to close this study at any time. The IEC and the competent regulatory authority must be informed within 15 days of early termination.

The trial or single dose steps will be terminated prematurely in the following cases:

- If adverse events occur which are so serious that the risk-benefit ratio is not acceptable.
- If the number of dropouts is so high that proper completion of the trial cannot realistically be expected.

8 METHODOLOGY

This is a single-center, pilot study (phase IIa) designed to evaluate the safety, feasibility, and efficacy of FMT from anti-PD-(L)1 responders or healthy donors to patients with HCC who failed to achieve or maintain a response to atezolizumab/bevacizumab. All patients admitted to the outpatient clinic or inpatient ward who are eligible will be informed about this pilot study. Patients will be included in this study, after having given written informed consent. Eligible patients will continue to receive atezolizumab/bevacizumab at the approved standard dose every 3 weeks after FMT. The study will enroll a total of 12 patients at the Division of Gastroenterology & Hepatology, Medical University of Vienna. Tumor response will be evaluated by radiological imaging which will take place after the first 3 cycles of

atezolizumab/bevacizumab following FMT and after every four cycles thereafter. Side effects will be evaluated continuously at every scheduled and unscheduled visit according to the CTCAE version 5.0. Treatment with atezolizumab/bevacizumab will continue until disease progression or unacceptable toxicity, whichever occurs first (Figure 2). Thereafter, information of progression, survival, and subsequent anti-cancer therapies will be collected regularly until death.

Patients who interrupt or permanently discontinue bevacizumab for adverse events are allowed to continue on single-agent atezolizumab. If atezolizumab is discontinued, bevacizumab will be discontinued as well.

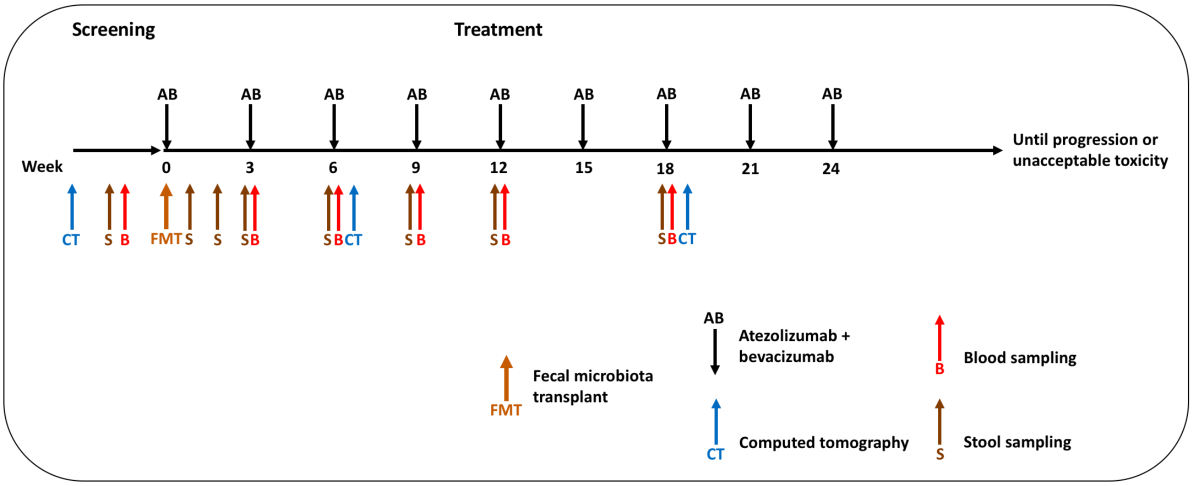

Figure 2 Study schema.

8.1 Study medication

The investigational medicinal products (IMPs) used in this pilot study are atezolizumab and bevacizumab.

Atezolizumab (trade name: Tecentriq, manufacturer: Roche), is an Fc-engineered, humanized IgG1 anti-programmed death-ligand 1 (PD-L1) monoclonal antibody produced in Chinese hamster ovary cells by recombinant DNA technology.[21] It will be supplied by the Sponsor as a sterile liquid in a single-use, 20-mL glass vial. The vial contains 20mL (1200mg) of atezolizumab solution. **Bevacizumab** (trade name: Avastin, manufacturer: Roche), a recombinant humanized monoclonal antibody against vascular endothelial growth factor (VEGF) produced by DNA technology in Chinese Hamster Ovary cells, will also be supplied by the Sponsor as a sterile liquid in single-use, 16-mL, preservative-free glass vials that contain 400mg of bevacizumab (25mg/mL).[22]

Atezolizumab will be administered first, followed by bevacizumab with a minimum of 5 minutes between the dosing.

8.1.1 Dosage and administration of Atezolizumab

Atezolizumab will be administered by IV infusion at a fixed dose of 1200 mg on day 1 and then every 21 days. No dose modification is allowed for atezolizumab. Treatment duration will last until loss of clinical benefit or unmanageable toxicity.

Atezolizumab	
**First infusion**	**Subsequent infusions**
• No premedication prior to the first atezolizumab infusion<br>• Vital signs (blood pressure, pulse rate, respiratory rate, temperature) should be measured within 60 minutes prior to the first infusion<br>• IV infusion over **60 (±15) minutes**<br>• If clinically indicated, vital signs can be measured during and after the infusion<br>• Patients should be instructed about the possibility of delayed post-infusion symptoms and actions to be taken (contact study physician) if they develop such symptoms	• If the patient experienced an infusion-related reaction with any previous infusion, premedication (antihistamines, antipyretics, and/or analgesics) may be administered at the discretion of the investigator<br>• Vital signs (blood pressure, pulse rate, respiratory rate, temperature) should be measured within 60 minutes prior to the infusion<br>• IV infusion over **30 (±10) minutes** if the previous infusion was tolerated without infusion-related reaction, or over **60 (±15) minutes** of the patient experienced an infusion-related reaction<br>• Vital signs should be measured during and after the infusion if the patient experienced an infusion-related reaction with the previous infusion or if clinically indicated

Table 3 Atezolizumab infusion details.

8.1.2 Dosage and administration of Bevacizumab

Bevacizumab will be administered by IV infusion at a dose of 15mg/kg on day 1 and then every 21 days. No dose modification is allowed for bevacizumab. Treatment duration will last until loss of clinical benefit or unmanageable toxicity.

Bevacizumab	
**First infusion**	**Subsequent infusions**
• No premedication prior to the first bevacizumab infusion<br>• Vital signs (blood pressure, pulse rate, respiratory rate, temperature) should be measured within 60 minutes prior to the first infusion<br>• IV infusion over **90 (±15) minutes**<br>• Vital signs should be measured at the end and 2 (±1) hours after the infusion<br>• Patients should be instructed about the possibility of delayed post-infusion symptoms and actions to be taken (contact	• If the patient experienced an infusion-related reaction with any previous infusion, premedication (antihistamines, antipyretics, and/or analgesics) may be administered at the discretion of the investigator<br>• Vital signs (blood pressure, pulse rate, respiratory rate, temperature) should be measured within 60 minutes prior to the infusion<br>• IV infusion over **60 (±10) minutes** if the previous infusion was tolerated without infusion-related reaction, or over **90 (±15) minutes** of the patient experienced an

study physician) if they develop such symptoms	infusion-related reaction. If the 60-minute infusion is well tolerated, bevacizumab may be infused over **30 (±15) minutes** thereafter<br>• Vital signs should be measured during and after the infusion if the patient experienced an infusion-related reaction with the previous infusion or if clinically indicated

Table 4 Bevacizumab infusion details.

8.1.3 Study drug interruption or discontinuation

The Investigator must temporarily interrupt or permanently discontinue the study drug if continued administration of the study drug is believed to be contrary to the best interests of the patient.

The interruption or premature discontinuation of study drug might be triggered by an AE, a diagnostic or therapeutic procedure, an abnormal assessment (e.g., laboratory abnormalities), or for administrative reasons, in particular withdrawal of the patient's consent.

The reason for study drug interruption or premature permanent discontinuation must be documented in the CRF.

8.1.4 Study drug interruption

Atezolizumab and bevacizumab treatment may be temporarily interrupted in patients experiencing toxicity considered to be related to study treatment.

Atezolizumab: If corticosteroids are initiated for treatment of the toxicity, they must be tapered over ≥1 month to the equivalent of ≤10mg/day oral prednisone before atezolizumab may be resumed. Atezolizumab may be withheld for >12 weeks to allow for patients to taper off corticosteroids prior to resuming treatment, if the investigator feels that the patient is likely to derive clinical benefit. **Bevacizumab**: If the event resolves to Grade ≤1, the treatment may be restarted at the same dose level. If any of the two treatments is delayed due to toxicity for >12 weeks (except patients who are tapered off corticosteroids), the patient must be permanently discontinued from the treatment. If atezolizumab is discontinued, bevacizumab should also be discontinued. If bevacizumab is discontinued, atezolizumab can be continued.

8.1.5 Study drug premature permanent discontinuation

The Investigator must permanently discontinue the study drug if continued administration of the study drug is believed to be contrary to the best interests of the patient. The premature discontinuation of study drug might be triggered by an AE, a diagnostic or therapeutic procedure, an abnormal assessment (e.g., laboratory abnormalities), or for administrative reasons, in particular withdrawal of the patient's consent. The reason for premature permanent discontinuation must be documented in the eCRF.

8.1.6 Study-drug delivery & drug storage conditions

All IMPs required for this study will be provided by the Sponsor and will be delivered to, stored and prepared by the local pharmacy according to the manufacturer's instructions. All IMPs will be prepared, labeled, and packed by the pharmacy according to local legal requirements and GMP.

8.1.7 IMP administration & handling

IMP administration will be performed as described in sections 8.1.1 and 8.1.2.

8.1.8 Drug accountability

IMPs will be provided by the sponsor and will be shipped to the study site. The study site will acknowledge receipt of IMPs supplied to confirm the shipment condition and content. Damaged shipments will be replaced. Drug Accountability, including received, dispensed, returned, and disposed IMPs, will be recorded at on-going basis. IMPs will be disposed of at the study site according to the study site's institutional standard operating procedure.

8.1.9 Procedures to assess subjects' compliance

Since the medication is given by IV infusion, compliance is guaranteed. Any deviation will be recorded in the eCRF.

8.1.10 Concomitant medication

Concomitant therapy consists of any medication (e.g., prescription drugs, over-the-counter drugs, vaccines, herbal or homeopathic remedies, nutritional supplements) used by study participants from the time of enrollment until the treatment/surveillance discontinuation visit in addition to the investigational drug. All concomitant medication should be reported to the investigator and recorded in the concomitant medications eCRF. Systemic corticosteroids (unless in low-dose or as one-time pulse dose e.g., for contrast allergy), immunosuppressive medications and TNF-α inhibitors should be used with caution at the discretion of the investigator. Any concomitant therapy intended for the treatment of cancer is prohibited.

Allowed: oral contraceptives, hormone-replacement therapy, inactivated influenza vaccinations, corticosteroids administered for chronic obstructive pulmonary disease or asthma, low-dose corticosteroids, low-dose aspirin

Not allowed: other anti-cancer treatments, live attenuated vaccines, systemic immunostimulatory agents (IFNs, IL-2)

8.2 Study procedures

The schedule of activities to be performed is provided in Table 1. All activities should be performed and documented for each patient. Patients will be closely monitored for safety and tolerability and will be assessed for toxicity prior to each dose. Dosing will only occur if the clinical assessment and laboratory values are acceptable.

8.2.1 General rules for trial procedures

- All study measures like blood sampling and measurements (vital parameters, ECG, etc.) have to be documented with date (dd.mm.yyyy).
- In case several study procedures are scheduled at the same time point, there is no specific sequence that should be followed.
- The dates of all procedures should be according to the protocol. The time margins mentioned in the study flow chart are admissible. If for any reason, a study procedure is not performed within scheduled margins, the procedure should be performed as soon as possible or as adequate.
- If it is necessary for organizational reasons, it is admissible to perform procedures which are scheduled for one visit at two different time points. Allowed time margins should thereby not be exceeded.

8.2.2 Screening investigation

Written informed consent for participation in the study must be obtained before performing any study-related procedures. Screening evaluations (for a detailed list see Table 1) must be completed and reviewed by study site staff to confirm that patients meet all eligibility criteria before study enrollment.

8.2.3 Study assessments

All necessary study procedures will be performed at the Medical University of Vienna.

Medical history (including surgeries, cancer history, reproductive status, smoking history, alcohol, and drugs use) and **concomitant medication** will be recorded at baseline. A complete **physical examination** will be performed at baseline and any abnormalities identified will be recorded on the general medical history and baseline conditions eCRF. If clinically indicated, symptom-directed physical examinations will be performed at specified visits.

Vitals signs, including measurements of pulse rate, systolic and diastolic blood pressure, and temperature, will be performed at each visit. Abnormalities at baseline will be recorded on the general medical history and baseline conditions eCRF. Clinically significant abnormalities observed during subsequent study visits will be recorded on the adverse event eCRF.

An **electrocardiogram (ECG)** is required at screening and when clinically indicated. ECG recordings must be reviewed, signed, and dated by the investigator and paper copies of ECG tracings must be kept at the study site file.

8.2.4 Laboratory assessments

Samples for the following **laboratory tests** will be sent to the local laboratory for analysis:

- Hematology: WBC count (incl. differential count, i.e., neutrophils, eosinophils, basophils, monocytes, lymphocytes, other cells), RBC count, hemoglobin, hematocrit, platelet count
- Chemistry panel (serum/plasma): sodium, potassium, chloride, phosphate, calcium, glucose, BUN or urea, creatinine, total protein, albumin, total bilirubin, ALP, ALT, AST, GGT, and LDH
- Coagulation: thromboplastin time (Owen), INR, and aPTT
- Hormone panel: TSH, fT3, fT4
- Tumor marker: AFP
- Virology: HIV serology, HBV serology (HBsAg, HBcAb, HBV DNA), HCV serology (HCV antibody, and if positive then HCV RNA)
- C-reactive protein
- Pregnancy test (all women of childbearing potential will have a serum pregnancy test within 14 days before Day 1 of Cylce 1): Urine pregnancy tests will be performed at specified visits. If a urine pregnancy test is positive, it must be confirmed by a serum pregnancy test.
- Urine analysis: dipstick (pH, blood, leukocytes, protein, ketones, glucose)
- Tumor tissue samples obtained at the time of HCC diagnosis will be used for exploratory research on biomarkers (if available)
- Gut tissue samples will be obtained at the time of colonoscopy and will be used for exploratory research including the analysis of the effect of FMT on the immune activity of the gut
- Additional exploratory analysis will include metagenomic and functional microbiota analysis before and after FMT, single cell analyses of circulating immune cells before and after FMT as well as serum proteomics, metabolomics and lipidomics signatures before and after FMT

8.2.5 Fecal microbiota transplant (FMT)

Fecal microbiota transplantation (FMT) is still a novel treatment modality and therefore, data about the most efficient way of performing FMT is scarce. Our study will be conducted according to the Austrian consensus [23] as well as the European guidelines on FMT [17].

Donor selection criteria

Potential donors will be patients with unresectable HCC treated with anti-PD-(L)1-based immunotherapy with complete or partial response for at least 12 months or healthy donors. In total, we are planning to include 2-3 donors, in whom infectious agents in the stool were ruled out by repetitive stool sampling.

Donor-specific exclusion criteria include history of antibiotic treatment within 2 months preceding donation; history of intrinsic gastrointestinal illnesses including inflammatory bowel disease, irritable bowel syndrome, chronic diarrhea (i.e., celiac disease), active primary gastrointestinal malignancies, or major gastrointestinal surgical procedures; history of symptomatic autoimmune illness; history of documented chronic pain syndromes (fibromyalgia, chronic fatigue) or neurologic and neurodevelopmental disorders; and history of metabolic syndrome, severe obesity (body mass index > 35), or moderate-to-severe malnutrition (as assessed clinically). Donors who tested positive for infections with SARS-CoV-2, latent potential (human immunodeficiency virus, hepatitis B virus, hepatitis C virus [only if PCR positive], human T-cell lymphotropic virus type 1 [HTLV-1], HTLV-2, strongyloides, syphilis) and/or had evidence of multi-drug resistant organisms such as vancomycin- resistant *Enterococcus*, carbapenem-resistant *Enterobacteriaceae*, and extended spectrum beta-lactamase are ineligible. Additionally, donors with FMT-relevant severe immune related adverse events (irAE), including colitis and hepatitis, are excluded.

Initial serological testing will be performed within 14 days prior to donor stool acquisition. Processed donor stool will be tested for the respective targets by applying the below listed methods (Table 5). Donors repeatedly used for FMT will be retested every 6 months or sooner, in the case of certain risks for infectious diseases.[13] To rule out SARS-CoV-2, fecal probes will be stored separately until 14 days after reception, to see if the donor might develop SARS-CoV-2 infection.

	**Target**	**Test**
**Bacteria**	ESBL, VRE, multidrugresistant gramnegative rods	Stool culture
	C.diff. (+toxin)	Stool culture, ELISA
	EAEC, EPEC, ETEC, EHEC, EIEC, tox C.diff, Campylobacter spp, Plesiomonas shigelloides, Salmonella spp., Vibrio parahaemolyticus, Vibrio vulnificus, Vibrio cholerae, Yersinia enteroloytica	Stool multiplex PCR
	Treponema pallidum	Serologic TPPA, TPHA
	Tbc	Serologic Quantiferon
**Parasites**	Cryptosporidium spp, Cyclospora cayetanensis, Entamoeba histolytica, Giardia lamblia	Stool multiplex PCR
	Ascariosis, Echinococcosis, Fasciolosis, Strongyloidosis, Toxocariosis, Trichinellosis, Cysticercosis, Trypanosoma cruzei/brucei	Serological testing
	Worms, Microspora, Protozoa, Helminths, Entamoeba histolytica, Lamblia giardia, Cryptospora, Cyclospora, Isospora	Stool microscopy, SAF fixation, Antigen detection, Ziehl-Neelsen stain

**Viruses**	Adenovirus F40/41; Astrovirus, Norovirus GI/GII, Rotavirus A; Sapovirus (I/II/IV/V)	Stool multiplex PCR
	Hepatitis A, B, C, D, E; HIV; CMV, HSV1+2, EBV, VZV, HHV6, HTLV 1+2; JC-Virus	Serological testing, PCR, Antibody detection

Table 5 Targets and methods of stool analyses.

Stool sample collection and DNA extraction

Stool samples are collected using a kit composed of sterile 30mL stool container, a sampling diaper and a cooler bag. After the collection of the multidonors' fecal probes for FMT, recipient stools are collected 7 days prior to the initiation of the depleting phase and repeatedly thereafter [17]. Patients are requested to sample their own stool at home, up to 24 hours before their arrival at the clinic. The containers are frozen in the patients' home freezer (-20°C) and transported to the clinic in a cooler bag. The containers are frozen upon receipt in a -80°C temperature monitored freezer.

Preparation of fecal material for FMT

Fecal material will be collected and prepared with an anaerobic preparation of the fecal probes [24 25]. Donor feces will be collected after the completion of the safety screening phase. Donor's feces are processed under GMP conditions and for the preparation process, regulations for the work with feces, classified as biohazard level 2, will be followed (wearing of water-repellent garments, gloves, facemasks, protective goggles, or shields). After the extraction of the fecal probe (approximately 50g of fecal matter for one colonoscopy-based implant), the entire donation will be diluted with 100–500 mL of sterile saline (0.9% NaCl) using 3x the weight of donor stool. Afterwards the mixture is homogenized in a sterilized blender or similar device, especially designated for this purpose. Afterwards the probe will sequentially be sieved to remove particulate material. The final slurry will be concentrated 3-fold by centrifugation.

Storage of donor fecal probes

Since instant application of fresh fecal probes to study recipients is not possible, the processed donor stool is mixed with glycerol (10% of total weight) and frozen at monitored temperature (-80°C). Before usage, the fecal probes will be gently reheated in a 37°C warm water bath over a period of 2 hours.

Patient microbiota depletion phase and application of fecal material by FMT

Patients will be prepared according to standard care with a colon lavage, routinely given prior to colonoscopy. To deplete the innate gut microbiota, patients receive an antibiotic treatment, according to protocols previously published [12,23,26], prior to colonoscopy consisting of an oral antibiotic treatment with vancomycin 500mg and neomycin 1000mg q6h for 72 hours.

For the application process, the Austrian consensus paper [23] recommends the lower gastrointestinal tract as the preferable route. The colonoscopy will be performed by an experienced endoscopist, and the procedure does not alter from standard colonoscopies, except for the administration of the fecal probes. During bowel intubation with the colonoscope residual stool will be suctioned to achieve maximal mucosal cleansing. After reaching 20cm into the terminal ileum, a 100mL fecal implant suspension will be administered via a catheter in the following sequence: 20mL in the terminal ileum, 40mL in the cecum and right colon, 20mL in the transverse colon and 20mL in the descending colon. After procedure completion, the recipients remain lying on prone Trendelenburg position for at least 4 hours to maintain the FMT suspension in the bowel. The day of the colonoscopy-based FMT is considered as day 0 of the trial protocol [12].

In patients with insufficient bowel lavage (i.e. patients with significant amounts of residual stool), colonoscopy will be prematurely terminated (due to an increased risk for bowel perforation), bowel lavage will be repeated and a second colonoscopy will be scheduled.

Complications and risks of FMT

Complications and risks vary and can range from flatulence and abdominal distension to fever, bacteremia, and perforation - similar to complications seen in standard colonoscopy. An additional risk is inadvertent transmission of pathogens. In line with current guidelines [17], the risk of transmission is minimalized using a multitude of serological and stool tests. Furthermore, potential donors with a history of gastrointestinal diseases and active chronic infectious diseases etc. (see above) are excluded. Another risk might be that recipients develop an irAE after FMT. However, there is currently no data to support this. On the contrary, FMT has been used several times to treat refractory irAE colitis [27,28].

8.2.6 Imaging assessments

Imaging assessments will be performed at screening, at the 3rd cycle and then every 4th cycle thereafter. Imaging assessments should be performed regardless of treatment delays and until disease recurrence or the end of the study, whichever occurs first. Screening assessments should include CT scans with IV contrast or MRI scans of chest, abdomen, and pelvis. All post-baseline imaging assessments will include CT scans with IV contrast or MRI scans of the chest, abdomen, and pelvis. The same radiographic procedures as used at screening should be used for subsequent imaging assessments and should be performed by the same evaluator, if possible, to ensure consistency across visits. Radiological response will be assessed according to mRECIST and RECIST v1.1 criteria.

8.2.7 Patient-reported outcomes instruments

Patient-reported outcome data will be obtained through use of the EQ-5D-5L questionnaire to fully characterize the clinical profile of atezolizumab and bevacizumab together with FMT. The EQ-5D-5L (EuroQol Group 1990; Brooks 1996; Herdman et al. 2011; Janssen et al. 2013) is a validated self-report health status questionnaire, designed to capture the patient's current health status. It comprises two components: a five-item health state profile that assesses mobility, self-care, usual activities, pain/discomfort, and anxiety/depression, as well as a visual analog scale (VAS) that measures health state. The EQ-5D-5L takes approximately 3 minutes to complete and will be used in this study.

8.2.8 End-of-study (EOS) examination

If patients are no longer eligible to participate in the study, patients undergo the end-of-study examination as listed in Table 1.

8.2.9 Definition of the end of the trial

The end of the study is defined as the date when the last patient enrolled had a follow-up of at least 20 weeks (2 scheduled follow-up imaging visits), if not progressed or deceased earlier.

9 SAFETY DEFINITIONS AND REPORTING REQUIREMENTS

9.1 Averse events (AEs)

9.1.1 Summary of known and potential risks associated with Atezolizumab

Atezolizumab has been associated with risks such as the following: infusion-related reactions (IRRs) and immune-mediated hepatitis, pneumonitis, colitis, pancreatitis, diabetes mellitus, hypothyroidism, hyperthyroidism, adrenal insufficiency, hypophysitis, Guillain-Barré syndrome, myasthenic syndrome or myasthenia gravis, meningoencephalitis, myocarditis, nephritis, myositis, and severe cutaneous adverse

reactions. Immune-mediated reactions may involve any organ system and may lead to hemophagocytic lymphohistiocytosis (HLH) and macrophage activation syndrome (MAS), which are considered to be potential risks for atezolizumab. Refer to Section 6 of the Atezolizumab Investigator's Brochure for a detailed description of anticipated safety risks for atezolizumab.

9.1.2 Summary of known and potential risks associated with Bevacizumab

Bevacizumab has been associated with risks such as the following: GI perforations, gallbladder perforation, hemorrhage, pulmonary hemorrhage, arterial thromboembolic events (ATE), fistulae, wound-healing complications, hypertension, congestive heart failure (CHF), cardiac disorders (excluding CHF and ATE), neutropenia, infections, necrotizing fasciitis, thrombocytopenia, venous thromboembolism, posterior reversible encephalopathy syndrome, pulmonary hypertension, ovarian failure, embryo-fetal development disturbance, hypersensitivity reactions/infusion reactions, peripheral sensory neuropathy, osteonecrosis of the jaw, non-mandibular osteonecrosis, thrombotic microangiopathy, and proteinuria. Refer to Section 6 of the Bevacizumab Investigator's Brochure for a detailed description of anticipated safety risks for Bevacizumab.

9.1.3 Definition of adverse events

An AE is any untoward adverse change from the subject's baseline condition, i.e., any unfavorable and unintended sign including an abnormal laboratory finding, symptom or disease which is considered to be clinically relevant by the physician that occurs during the course of the study, whether or not considered related to the study drug.

Adverse events include:

- Exacerbation of a pre-existing disease.
- Increase in frequency or intensity of a pre-existing episodic disease or medical condition.
- Disease or medical condition detected or diagnosed after study drug administration even though it may have been present prior to the start of the study.
- Continuous persistent disease or symptoms present at baseline that worsen following the start of the study.
- Lack of efficacy in the acute treatment of a life-threatening disease.
- Events considered by the investigator to be related to study-mandated procedures.
- Abnormal assessments, e.g., ECG and physical examination findings, must be reported as AEs if they represent a clinically significant finding that was not present at baseline or worsened during the course of the study.
- Laboratory test abnormalities must be reported as AEs if they represent a clinically significant finding, symptomatic or not, which was not present at baseline or worsened during the course of the study or led to dose reduction, interruption or permanent discontinuation of study drug.

Adverse events do not include:

- Pre-planned interventions or occurrence of endpoints specified in the study protocol are not considered AE´s, if not defined otherwise (e.g., as a result of overdose)
- Medical or surgical procedure, e.g., surgery, endoscopy, tooth extraction, transfusion. However, the event leading to the procedure is an AE. If this event is serious, the procedure must be described in the SAE narrative.
- Pre-existing disease or medical condition that does not worsen.
- Situations in which an adverse change did not occur, e.g., hospitalizations for cosmetic elective surgery or for social and/or convenience reasons.
- Overdose of either study drug or concomitant medication without any signs or symptoms. However, overdose must be mentioned in the Study Drug Log.

9.1.4 Adverse event reporting period

Investigators will seek information on adverse events at each patient contact (at clinic visit or using telemedicine). All adverse events, whether reported by the patient or noted by study personnel, will be recorded in the patient's medical record and on the Adverse Event eCRF.

9.2 Serious adverse events (SAEs)

A Serious Adverse Event (SAE) is defined by the International Conference on Harmonization (ICH) guidelines and GCP guidelines as any AE fulfilling at least one of the following criteria:

- Results in deaths.
- Life-threatening – defined as an event in which the subject was, in the judgment of the investigator, at risk of death at the time of the event;
- Requiring subject's hospitalization or prolongation of existing hospitalization
- Resulting in persistent or significant disability or incapacity (i.e., a substantial disruption of a person's ability to conduct normal life functions).
- Congenital anomaly or birth defect.
- Optional: Is medically significant or requires intervention to prevent at least one of the outcomes listed above

Life-threatening refers to an event in which the subject was at risk of death at the time of the event. It does not refer to an event that hypothetically might have caused death if it were more severe.

Important medical events that may not immediately result in death, be life-threatening, or require hospitalization may be considered as SAEs when, based upon appropriate medical judgment, they may jeopardize the subject and may require medical or surgical intervention to prevent one of the outcomes listed in the definitions above. This means an individual case decision.

9.2.1 Hospitalization – Prolongation of existing hospitalization

Hospitalization is defined as an overnight stay in a hospital unit and/or emergency room.

An additional overnight stay defines a prolongation of existing hospitalization.

The following is not considered an SAE and should be reported as an AE only:

- Treatment on an emergency or outpatient basis for an event not fulfilling the definition of seriousness given above and not resulting in hospitalization.

The following reasons for hospitalizations are not considered AEs, and therefore not SAEs:

- Hospitalizations for cosmetic elective surgery, social and/or convenience reasons.
- Elective treatment of a pre-existing disease or medical condition that did not worsen, e.g., hospitalization for chemotherapy for cancer, elective hip replacement for arthritis.

9.2.2 SAEs related to investigational drug

Such SAEs are defined as SAEs that appear to have a reasonable possibility of causal relationship.

9.3 Suspected unexpected serious adverse reactions (SUSARs)

SUSARs are all serious adverse reactions with **suspected** causal relationship to the study drug that is **unexpected** (not previously described in the Summary of Product Characteristics or Investigator's brochure) and serious.

9.4 Adverse events of special interest (AESI; immediately reportable to the sponsor)

Adverse events of special interest are required to be reported by the investigator to the sponsor immediately (i.e., no more than 24 hours after learning of the event; see Section 9.6.3 for reporting instructions). Adverse events of special interest for this study are as follows:

- Cases of potential drug-induced liver injury that include an elevated ALT or AST (>3 x baseline value) in combination with either an elevated bilirubin (>2 x ULN) or clinical jaundice, as defined by Hy's Law
- Suspected transmission of an infectious agent by a study treatment, as defined below:

  Any organism, virus, or infectious particle (e.g., prion protein transmitting transmissible spongiform encephalopathy), pathogenic or non-pathogenic, is considered an infectious agent. A transmission of an infectious agent may be suspected from clinical symptoms or laboratory findings that indicate an infection in a patient exposed to a medicinal product. This term applies only when a contamination of the study treatment is suspected.

- Pneumonitis
- Colitis
- Endocrinopathies (e.g. diabetes mellitus, pancreatitis, adrenal insufficiency, hyperthyroidism, hypophysitis)
- Hepatitis, including AST or ALT >10 x ULN
- Systemic lupus erythematosus
- Neurological disorders (e.g. Guillan-Barré syndrome, myasthenic syndrome or myasthenia gravis, meningoencephalitis)
- Events suggestive of hypersensitivity, infusion-related reactions, cytokine release syndrome, HLH, and MAS
- Nephritis
- Ocular toxicities (e.g., uveitis, retinitis, optic neuritis)
- Myositis and myopathies, including rhabdomyolysis
- Grade ≥ 2 cardiac disorders (e.g. atrial fibrillation, myocarditis, pericarditis)
- Vasculitis
- Autoimmune hemolytic anemia
- Severe cutaneous reactions (e.g. Stevens-Johnson syndrome, dermatitis bullous, toxic epidermal necrolysis)
- Grade ≥3 hypertension
- Grade ≥3 proteinuria
- Any grade GI perforation, abscess, or fistula
- Grade ≥2 non-GI fistula or abscess
- Grade ≥3 wound-healing complication
- Any arterial thromboembolic event
- Hemorrhage (e.g. grade ≥2 hemoptisis, other grade ≥3 hemorrhagic event)
- Grade ≥3 venous thromboembolic event
- Grade ≥3 chronic heart failure

9.5 Severity of adverse events

The adverse event severity grading scale for the National Cancer Institute (NCI) Common Terminology Criteria for Adverse Events (CTCAE) version 5.0 will be used for assessing adverse event severity. Table 5 will be used for assessing severity for adverse events that are not specifically listed in the NCI CTCAE (v5.0).

Grade	Severity
1	Mild; asymptomatic or mild symptoms; clinical or diagnostic observations only; or intervention not indicated
2	Moderate; minimal, local, or non-invasive intervention indicated; or limiting age-appropriate instrumental activities of daily living [a]
3	Severe or medically significant, but not immediately life-threatening; hospitalization or prolongation of hospitalization indicated; disabling; or limiting self-care activities of daily living [b,c]
4	Life-threatening consequences or urgent intervention indicated [d]
5	Death related to adverse event [d]
	[a] Instrumental activities of daily living refer to preparing meals, shopping for groceries or clothes, using the telephone, managing money, etc.<br>[b] Examples of self-care activities of daily living include bathing, dressing and undressing, feeding oneself, using the toilet, and taking medications, as performed by patients who are not bedridden.<br>[c] If an event is assessed as a "significant medical event," it must be reported as a serious adverse event<br>[d] Grade 4 and 5 events must be reported as serious adverse events

Table 6 Grading scale for assessing severity of adverse events that are not specifically listed in the NCI CTCAE (v5.0). Based on the most recent version of NCI CTCAE (v5.0), which can be found at: http://ctep.cancer.gov/protocolDevelopment/electronic_applications/ctc.htm

If the severity of an AE worsens during study drug administration, only the worst intensity should be reported on the AE page. If the AE lessens in intensity, no change in the severity is required.

If an AE occurs during a washout or placebo run-in phase and afterwards worsens during the treatment phase, a new AE page must be filled in with the intensity observed during study drug administration.

9.6 Relationship to study drug

For all AEs, the Investigator will assess the causal relationship between the study drug and the AE using his/her clinical expertise and judgment according to the following algorithm that best fits the circumstances of the AE:

Not related

- May or may not follow a temporal sequence from administration of the study product
- Is biologically implausible and does not follow known response pattern to the suspect study drug (if response pattern is previously known).
- Can be explained by the known characteristics of the subject’s clinical state or other modes of therapy administered to the subject.

Unlikely

- There is a reasonable temporal relation between the AE and the intake of the study medication, but there is a plausible other explanation for the occurrence of the AE.

Possibly

- The AE has a reasonable temporal relationship with drug administration.
- The AE may equally be explained by the study subject’s clinical state, environmental or toxic factors, or concomitant therapy administered to the study subject.
- The relationship between study drug and AE may also be pharmacologically or clinically plausible.

Probably

- There is a reasonable temporal relation between the AE and the intake of the study medication, and plausible reasons point to a causal relation with the study medication.

Related

- Reasonable temporal relation between the AE and the intake of the study medication and
- There is no other explanation for the AE and
- Subsidence or disappearance of the AE on withdrawal of the study medication and
- Recurrence of the symptoms on restart at previous dose (only applies for re-institution of mediation).

9.7 Reporting procedures

A special section is designated to adverse events in the case report form. The following details must thereby be entered:

- Type of adverse event
- Start (date and time)
- End (date and time)
- Severity (mild, moderate, severe)
- Serious (no / yes)
- Unexpected (no / yes)
- Outcome (resolved, resolving, not resolved, resolved with sequelae, unknown, fatal)
- Relation to study drug (Related/ Probably/ Possibly/ Unlikely/ Not related)

Adverse events are to be documented in the case report form in accordance with the above mentioned criteria.

9.7.1 Reporting procedures for SAEs

In case of a serious adverse event, the investigator has to use all supportive measures for best patient treatment. A written report is also to be prepared and should at least contain the following:

- Patient number
- Patient: sex
- The suspected investigational medical product (IMP)
- The adverse event assessed as serious
- Short description of the event and outcome

If applicable, the initial report should be followed by the follow up report, indicating the outcome of the SAE.

9.7.2 Reporting procedures for SUSAR

It must be remembered that the regulatory authorities, and the Institutional Review Board / Independent Ethics Committee (IRB / IEC) must be informed about all SUSAR. Such reports shall be made by the sponsor and should content at least the following details:

- Patient number (study code/screening number)
- Patient: age in years, sex
- Name of Investigator and investigating site
- Period of administration
- The suspected investigational medical product (IMP)
- The adverse event assessed as serious and unexpected, and for which there is a **suspected** causal relationship to the IMP
- Concomitant disease and medication
- Short description of the event:
    - Description
    - Onset and if applicable, end
    - Therapeutic intervention

- Causal relationship
- Seriousness criteria or reportable reason

Electronic reporting should be the expected method for reporting of SUSARs to the competent authority. In that case, the format and content as defined by the regulatory requirements should be adhered to. The latest version of MedDRA should be applied. Lower level terms (LLT) should be used.

9.7.3 Reporting procedures for AESIs

After initiation of study treatment on Day 1 of Cycle 1, serious adverse events and adverse events of special interest will be reported until 90 days after the final cycle or until initiation of new systemic anti-cancer therapy, whichever occurs first. For adverse events of special interest in patients receiving active treatment and for serious adverse events in all patients, investigators should record all case details that can be gathered immediately (i.e., within 24 hours after learning of the event) on the Adverse Event eCRF and submit a report to the sponsor.

9.7.4 Reporting procedures for pregnancies

Any pregnancy that occurs during study participation must be reported to the investigator/sponsor. To ensure subject safety, each pregnancy must be reported to the sponsor immediately. The pregnancy must be followed up to determine outcome (including premature termination) and status of mother and child. Pregnancy complications and elective terminations for medical reasons must be reported as an AE or SAE. Spontaneous abortions must be reported as an SAE.

9.7.4.1 Pregnancies in female patients

Female patients of childbearing potential will be instructed through the Informed Consent Form to immediately inform the investigator/sponsor if they become pregnant during the study or within 5 months after the final dose of atezolizumab or 6 months after the final dose of bevacizumab. Pregnancy should not be recorded on the Adverse Event eCRF. The investigator should discontinue study treatment and counsel the patient, discussing the risks of the pregnancy and the possible effects on the fetus. Monitoring of the patient should continue until conclusion of the pregnancy. Any serious adverse events associated with the pregnancy (e.g., an event in the fetus, an event in the mother during or after the pregnancy, or a congenital anomaly/birth defect in the child) should be reported on the Adverse Event eCRF.

9.7.4.2 Pregnancies in female partners of male patients

Male patients will be instructed through the Informed Consent Form to immediately inform the investigator/sponsor if their partner becomes pregnant during the study or within 6 months after the final dose of bevacizumab. Attempts should be made to collect and report details of the course and outcome of any pregnancy in the partner of a male patient exposed to study treatment. An investigator who is contacted by the male patient or his pregnant partner may provide information on the risks of the pregnancy and the possible effects on the fetus, to support an informed decision in cooperation with the treating physician and/or obstetrician.

10 FOLLOW-UP

10.1 Follow-up of study participants including follow-up of adverse events

After study termination for whatever reason, patients will receive one protocol defined follow-up visit within the study, followed by regular follow-up visits according to standards of clinical routine thereafter. Furthermore, routine follow-up imaging assessments will be performed, and the investigator should

follow each adverse event until the event has resolved to baseline grade or better, the event is assessed as stable by the investigator, the patient is lost to follow-up, or the patient withdraws consent.

10.2 Treatment after end of study

No treatment is described after the end of the study. The post study treatment depends on the individual problems of each patient and will be decided by the treating physician.

11 STATISTICAL METHODOLOGY AND ANALYSIS

In brief, only descriptive statistics will be used to evaluate the primary objective.

Data will be obtained according to the visit and assessment schedule as outlined above. Source data documentation will be performed using the electronic patient management software of the General hospital of Vienna (AKIM) and an electronic case report form (eCRF) that was generated for this study using the Clincase-software will be used for data management. Monitoring and source data verification will be performed by an external CRO.

Due to the exploratory study design, no formal power analysis has been performed. However, the number of patients planned to be included into this pilot study is expected to be sufficient to fulfill the study's objectives.

The level of significance will be defined at a p-value <0.05.

11.1 Analysis sets

For the analysis of the primary safety objective, all subjects who underwent technically successful fecal microbiota transplantation and received the study drug (at least one dose) and did not violate the protocol in a way that might affect the evaluation of the effect of the combination of FMT and the study drugs on the primary objective, i.e., without major protocol violations, will be included. Patients who do not undergo FMT will be replaced. For the secondary objectives and exploratory endpoints, two different analysis sets will be used.

Intention to treat set

This analysis set includes all subjects. Therefore, this analysis set also includes patients in whom FMT was not (successfully) performed for whatever reason as well as patients who did not receive the study drugs.

Per-protocol set

This analysis set comprises all subjects who underwent successful FMT, received the study drugs (at least one dose) and did not violate the protocol in a way that might affect the evaluation of the effect of the study drug(s) on the primary objective, i.e., without major protocol violations.

11.2 Sample size considerations

For this clinical trial no inferential statistical testing is planned and therefore an exact sample size requirement has not been specified. The number of patients planned to be included is expected to be sufficient to fulfill the pilot study's objectives. Previous studies evaluating the efficacy of FMT in patients with advanced melanoma not responding to immunotherapy evaluated a comparable number of patients.[12,13]

11.3 Relevant protocol deviations

All protocol deviations will be listed in the study report.

Major protocol violations

Major protocol violations include the following situations:

- Any situations that hinder successful FMT (patients with insufficient bowel preparation, patients violating post-FMT-procedure without a second successful procedure performed)
- Patients taking antimicrobial substances shortly after FMT
- Patients being lost to follow-up prior to the first re-staging imaging examination
- Patients not receiving the study drugs after FMT for any reason

11.4 Endpoint analysis

11.4.1 Primary endpoint analysis

The primary endpoint will be analyzed using descriptive statistics. In detail, the incidence and severity of treatment-related adverse events determined according to National Cancer Institute (NCI) Common Terminology Criteria for Adverse Events (CTCAE) version 5.0 will be described.

11.4.2 Secondary endpoint analysis

Efficacy

Efficacy will be evaluated by the number (percentage) of study participants achieving complete response (CR), partial response (PR), stable disease (SD) or progressive disease (PD) as best radiological response evaluated according to mRECIST and RECIST v1.1 criteria. Objective response is defined as either complete or partial response, while disease control rate comprises complete/partial response as well as stable disease. Kaplan-Meier method will be used to calculate progression-free survival and overall survival.

Quality of life

Distribution of quality of life parameters will be assessed by plotting histograms. Baseline and follow-up values will be demonstrated as mean (+/- standard deviation) or median (IQR), as applicable. Changes of quality of life during the study period as assessed by EQ-5D-5L questionnaire will be evaluated using paired T-test or Wilcoxon signed rank test.

11.4.3 Exploratory objectives

Effect of FMT on recipient gut microbiota composition, diversity (alpha and beta), rate of change from baseline and similarity to donor stool composition over time as well as comparison of responders and non-responders

- Bacterial DNA is extracted from stool using the Stool mini kit (Qiagen) and amplicon sequencing of the 16S V3-V4 region will be performed using an established pipeline with MiSeq technology. Statistical analysis is done using R; to analyze the similarity of microbial profiles, UniFrac distances and bray cutis dissimilarity are used. DSEq2 is used to compare bacterial abundance. For functional pathway analysis and metagenome assemblies a shotgun metagenomic approach with Illumina NovaSeq technology will be applied on selected samples.[29]

Metagenome assemblies and functional profiling using shotgun metagenomic sequencing

- **Metagenome assemblies**: Genomes will be assembled using megahit and the assemblies will be binned using matabat2. Genome bins will be classified using GTDB and checked for completeness using CheckM. For analysis of beta diversity multidimensional scaling of UniFrac distances and PERMANOVA testing between groups will be performed. To compare relative abundances of bacterial strains DESeq2 will be used. Pearson's correlation between metabolites, immune cells and relative abundance of different bacterial strains will be calculated.

- **Functional pathway analysis**: For gene abundance estimation and per-species and community-level metabolic network reconstruction the HUMAnN 3.0 pipeline will be used. Differences in metabolic pathways will be analyzed between donor and recipient over time as well as responders vs. non-responders.

Serum and stool metabolomic and lipidomic signatures before and after FMT

Metabolomics data processing will be performed using commercial Agilent Mass Hunter and Thermo Scientific Trace Finder software, and R. Peak finding and alignment will be performed with Mass Hunter and Trace Finder software. The vendor data format will be converted to an open format using ProteoWizard for an independent analysis. Missing value imputation, noise and outlier filtering, and normalization will be performed with relevant Bioconductor packages before statistical analysis of the spectra data.

Statistical analysis of multivariate spectra data will be performed using principal component analysis (PCA) for data exploration and Orthogonal Projections to Latent Structures Discriminant Analysis (OPLS-DA). In the latter, variable influence on projection (VIP) ≥ 1, together with p-value ≤ 0.05 (t-test) and fold change ≥ 2 will be used as a feature selection threshold to identify metabolites associated with a condition, its severity, or with a patient subgroup.

Variable name	Categories	Variable type	Design
**Microbiome composition**	Downstream of bioinformatics pipeline [29]: Relative abundance matrix of bacterial amplicon sequence variants (ASVs)	Metric	Multidimensional scaling of UniFrac distances and PERMANOVA testing between groups, Kruskal-Wallis Rank Sum Test with Benjamini-Hochberg correction of relative abundances of ASVs, before and after FMT, between responders vs non-responders. Pearson's correlation between responders and relative abundances of ASVs
**Microbiome diversity**	Downstream of bioinformatics pipeline [29]: Shannon diversity indices	Metric	Mann Whitney U with Bonferroni correction:<br>Before and after FMT, between responders vs non-responders.
**Functional metabolomic profiling**	Untargeted metabolomics, lipids	Metric	Kruskal-Wallis Rank Sum Test with Benjamini-Hochberg correction: Before and after FMT, between responders vs non-responders. Pearson's correlation between responders and metabolites concentration
**Shotgun metagenomic sequencing**	Downstream of bioinformatics pipeline: Gene abundance estimation and per-species and community-level metabolic network	Metric	Multidimensional scaling of UniFrac distances and PERMANOVA testing between responders and non-responders. relative abundances of bacterial strains DESeq2 will be used.

	reconstruction using HUMAnN 3.0 pipeline		Pearson's correlation between metabolites, immune cells and relative abundance of different bacterial strains will be calculated.

Table 7 List and explanation of exploratory objectives.

11.5 Interim analysis

An interim analysis will be performed after six subjects (50% of the planned study population) underwent at least one radiological follow-up. If disease control (CR/PR/SD) as best radiological response according to mRECIST cannot be achieved in ≥50% (3 subjects) of the first 6 subjects, the study will be terminated prematurely as a disease control of >50% may be achieved with alternative second line treatment options.

11.6 Software program(s)

- Statistical analyses of all data: IBM SPSS Statistics 26 (SPSS Inc., Armonk, New York, USA), R
- Figures and graphical design: GraphPad Prism 8 (GraphPad Software, La Jolla, California, USA), R
- eCRF: Clincase

12 DOCUMENTATION AND DATA MANAGEMENT

12.1 Documentation of study results

A subject screening and identification Log will be completed for all enrolled subjects with the reasons for exclusion.

12.1.1 Case report form (CRF)

An electronic CRF will be used in this study.

For each subject enrolled, regardless of study drug initiation, a CRF must be completed and signed by the investigator or a designated sub-investigator. This also applies to those subjects who fail to complete the study. If a subject withdraws from the study, the reason must be noted on the CRF. Case report forms are to be completed on an ongoing basis.

Screening failures will not be documented in the CRF.

CRF entries and corrections will only be performed by study site staff, authorized by the investigator. The entries will be checked by trained personnel (monitor) and any errors or inconsistencies will be checked immediately.

12.1.2 Data collection

Data collected at all visits are entered into an interactive form. The CRFs will be source documents verified following guidelines established before study onset as detailed in the monitoring plan. Maintenance of the study database will be performed by study site staff.

12.2 Safekeeping

The Investigator will maintain adequate and accurate records to enable the conduct of the study to be fully documented and the study data to be subsequently verified (according to ICH-GCP "essential documents"). These documents will be classified into two different categories: investigator's study site file (ISF) with all essential documents regarding the study conduct, and subject clinical source documents.

The investigator's file will contain all essential documents listed in ICH-GCP Guidelines section 8.

Subject clinical source documents include all patient hospital clinical records in original version, such as original laboratory reports, ECG, X-ray prints and other reports.

These two categories of documents must be kept on file by the Investigator for as long as needed to comply with the regulatory requirements.

12.3 Quality control and quality assurance

The overall procedures for quality assurance of clinical trials are described in the Standard Operational Procedures (SOPs) of the Medical University of Vienna.

12.3.1 Periodic Monitoring

The designated monitor will contact and visit the investigator on a regularly basis and will be allowed to have direct access to all source documents needed to verify the entries in the CRFs and other protocol-related documents provided that subject confidentiality is maintained in agreement with local regulations. It will be the monitor's responsibility to inspect the CRFs at regular intervals according to the monitoring plan throughout the study, to verify the adherence to the protocol and the completeness, consistency and accuracy of the data being entered on them.

Three monitoring visits are planned: one at study initiation, one within 8 weeks of enrollment of the first participant and one during the close out visit. All (one-hundered %) of source data will be checked by the monitor.

12.3.2 Audit and inspections

Upon request, the investigator will make all study-related source data and records available to a qualified quality assurance auditor mandated by the sponsor or to competent authority inspectors. The main purposes of an audit or inspection are to confirm that the rights and welfare of the subjects have been adequately protected, and that all data relevant for assessment of safety and efficacy of the investigational product have appropriately been reported to the sponsor.

12.4 Reporting and publication

12.4.1 Publication of study results

The findings of this study will be published by the sponsor (investigators) in a scientific journal and presented at scientific meetings. The manuscript will be circulated to all co-investigators before submission. Confidentiality of subjects in reports/publications will be guaranteed.

13 ETHICAL AND LEGAL ASPECTS

13.1 Informed consent of subjects

Following comprehensive instruction regarding the nature, significance, impact and risks of this clinical trial, the patient must give written consent to participation in the study.

During the instruction the trial participants are to be made aware of the fact that they can withdraw their consent – without giving reasons – at any time without their further medical care being influenced in any way.

In addition to the comprehensive instructions given to the trial participants by the investigator, the trial participants also receive a written patient information sheet in comprehensible language, explaining the nature and purpose of the study and its progress.

The patients must agree to the possibility of study-related data being passed on to relevant authorities.

The patients must be informed in detail of their obligations in relation to the trial participants insurance in order not to jeopardize insurance cover.

13.2 Acknowledgement / approval of the study

The Investigator (or a designated CRO) will submit this protocol and any related document provided to the subject (such as subject information used to obtain informed consent) to the Ethics Committee (EC) of the Medical University of Vienna. Approval from the committee must be obtained before starting the study.

The clinical trial shall be performed in full compliance with the legal regulations according to the Drug Law (AMG - Arzneimittelgesetz) of the Republic of Austria.

An application must also be submitted to the Austrian Competent Authorities (Bundesamt für Sicherheit im Gesundheitswesen (BASG) represented by the Agency for Health and Food Safety (AGES Medizinmarktaufsicht) and registered to the European Clinical Trial Database (EudraCT) using the required forms.

13.2.1 Changes in the conduct of the study

Protocol amendments

Proposed amendments must be submitted to the appropriate CA and ECs. Substantial amendments may be implemented only after CA/EC approval has been obtained. Amendments that are intended to eliminate an apparent immediate hazard to subjects may be implemented prior to receiving CA/EC approval. However, in this case, approval must be obtained as soon as possible after implementation.

Study Termination

If the sponsor or the Investigator decides to terminate the study before the planned completion, they will notify each other in writing stating the reasons of early termination. Both the sponsor and the investigator will ensure the protection of the subjects' wellbeing. The sponsor will notify the regulatory authority as well as the ethics committee about the premature termination. Documentation will be filed in the Trial Master File as well as in the Investigator Site File.

Clinical Study Report (CSR)

Within one year after the final completion of the study, a full CSR will be prepared by the sponsor and submitted to the EC and the competent authority.

The Investigator will be asked to review and sign the final study report.

13.3 Insurance

During their participation in the clinical trial the patients will be insured as defined by legal requirements. The Investigator of the clinical trial will receive a copy of the insurance conditions of the 'patients insurance'. The sponsor is providing insurance (MedUni Wien-Rahmenversicherung klinischer Studien, Polizze-Nr. 07229622-2, lfd. Nr. 5/2022, Universitätsklinik für Innere Medizin III) in order to indemnify (legal and financial coverage) the investigator/center against claims arising from the study, except for claims that arise from malpractice and/or negligence. The compensation of the subject in the event of study-related injuries will comply with the applicable regulations.

Details on the existing patients insurance are given in the patient information sheet.

13.4 Confidentiality

The information contained in this document, especially unpublished data, is the property of the Medical University of Vienna. It is therefore provided to you in confidence as an investigator, potential investigator, or consultant, for review by you, your staff, and an Ethics Committee or Institutional Review

Board. It is understood that this information will not be disclosed to others without written authorization from the principal investigator.

13.5 Ethics and good clinical practice (GCP)

The Investigator will ensure that this study is conducted in full conformance with the principles of the "Declaration of Helsinki" (as amended at the 64th WMA General Assembly, Fortaleza, Brazil, 2013) and with the laws and regulations of the country in which the clinical research is conducted.

The Investigator of the clinical trial shall guarantee that only appropriately trained personnel will be involved in the study. All studies must follow the ICH GCP Guidelines and the regulatory requirements.

Therefore this study follows the EU Directive embedded in the Austrian drug act.

14 REFERENCES

1. European Association for the Study of the Liver. Electronic address eee, European Association for the Study of the L. EASL Clinical Practice Guidelines: Management of hepatocellular carcinoma. *J Hepatol*. Jul 2018;69(1):182-236. doi:10.1016/j.jhep.2018.03.019
2. Bruix J, Chan SL, Galle PR, Rimassa L, Sangro B. Systemic treatment of hepatocellular carcinoma: An EASL position paper. *J Hepatol*. Jul 10 2021;doi:10.1016/j.jhep.2021.07.004
3. Pinter M, Jain RK, Duda DG. The Current Landscape of Immune Checkpoint Blockade in Hepatocellular Carcinoma: A Review. *JAMA oncology*. Jan 1 2021;7(1):113-123. doi:10.1001/jamaoncol.2020.3381
4. Finn RS, Qin S, Ikeda M, et al. Atezolizumab plus Bevacizumab in Unresectable Hepatocellular Carcinoma. *N Engl J Med*. May 14 2020;382(20):1894-1905. doi:10.1056/NEJMoa1915745
5. Pinter M, Scheiner B, Peck-Radosavljevic M. Immunotherapy for advanced hepatocellular carcinoma: a focus on special subgroups. *Gut*. Jan 2021;70(1):204-214. doi:10.1136/gutjnl-2020-321702
6. Ducreux M, Zhu AX, Cheng AL, et al. IMbrave150: Exploratory analysis to examine the association between treatment response and overall survival (OS) in patients (pts) with unresectable hepatocellular carcinoma (HCC) treated with atezolizumab (atezo) + bevacizumab (bev) versus sorafenib (sor). *J Clin Oncol*. 2021;39 (15_suppl):4071-4071.
7. Schwabe RF, Greten TF. Gut microbiome in HCC - Mechanisms, diagnosis and therapy. *Journal of hepatology*. Feb 2020;72(2):230-238. doi:10.1016/j.jhep.2019.08.016
8. Behary J, Amorim N, Jiang XT, et al. Gut microbiota impact on the peripheral immune response in non-alcoholic fatty liver disease related hepatocellular carcinoma. *Nature communications*. Jan 8 2021;12(1):187. doi:10.1038/s41467-020-20422-7
9. Routy B, Le Chatelier E, Derosa L, et al. Gut microbiome influences efficacy of PD-1-based immunotherapy against epithelial tumors. *Science*. Jan 5 2018;359(6371):91-97. doi:10.1126/science.aan3706
10. Matson V, Fessler J, Bao R, et al. The commensal microbiome is associated with anti-PD-1 efficacy in metastatic melanoma patients. *Science*. Jan 5 2018;359(6371):104-108. doi:10.1126/science.aao3290
11. Gopalakrishnan V, Spencer CN, Nezi L, et al. Gut microbiome modulates response to anti-PD-1 immunotherapy in melanoma patients. *Science*. Jan 5 2018;359(6371):97-103. doi:10.1126/science.aan4236
12. Baruch EN, Youngster I, Ben-Betzalel G, et al. Fecal microbiota transplant promotes response in immunotherapy-refractory melanoma patients. *Science*. Feb 5 2021;371(6529):602-609. doi:10.1126/science.abb5920
13. Davar D, Dzutsev AK, McCulloch JA, et al. Fecal microbiota transplant overcomes resistance to anti-PD-1 therapy in melanoma patients. *Science*. Feb 5 2021;371(6529):595-602. doi:10.1126/science.abf3363
14. Lencioni R, Llovet JM. Modified RECIST (mRECIST) assessment for hepatocellular carcinoma. *Semin Liver Dis*. Feb 2010;30(1):52-60. doi:10.1055/s-0030-1247132
15. Finn RS, Qin A, Ikeda M, et al. Complete responses (CR) in patients receiving atezolizumab (atezo) + bevacizumab (bev) versus sorafenib (sor) in IMbrave150: A phase III clinical trial for unresectable hepatocellular carcinoma (HCC). *J Clin Oncol*. 2021;38 (15_suppl):4596-4596.
16. D'Haens GR, Jobin C. Fecal Microbial Transplantation for Diseases Beyond Recurrent Clostridium Difficile Infection. *Gastroenterology*. Sep 2019;157(3):624-636. doi:10.1053/j.gastro.2019.04.053
17. Cammarota G, Ianiro G, Tilg H, et al. European consensus conference on faecal microbiota transplantation in clinical practice. *Gut*. Apr 2017;66(4):569-580. doi:10.1136/gutjnl-2016-313017
18. Bruix J, Qin S, Merle P, et al. Regorafenib for patients with hepatocellular carcinoma who progressed on sorafenib treatment (RESORCE): a randomised, double-blind, placebo-controlled, phase 3 trial. *Lancet*. Jan 7 2017;389(10064):56-66. doi:10.1016/S0140-6736(16)32453-9
19. Abou-Alfa GK, Meyer T, Cheng AL, et al. Cabozantinib in Patients with Advanced and Progressing Hepatocellular Carcinoma. *The New England journal of medicine*. Jul 5 2018;379(1):54-63. doi:10.1056/NEJMoa1717002
20. Zhu AX, Kang YK, Yen CJ, et al. Ramucirumab after sorafenib in patients with advanced hepatocellular carcinoma and increased alpha-fetoprotein concentrations (REACH-2): a randomised, double-blind, placebo-controlled, phase 3 trial. *Lancet Oncol*. Feb 2019;20(2):282-296. doi:10.1016/S1470-2045(18)30937-9
21. Agency E-EM. Atezolizumab (Tecentriq) [summary of product characteristics]. accessed November 02, 2021, https://www.ema.europa.eu/en/documents/product-information/tecentriq-epar-product-information_en.pdf
22. Agency E-EM. Bevacizumab (Avastin) [summary of product characteristics]. accessed November 02, 2021, https://www.ema.europa.eu/en/documents/product-information/avastin-epar-product-information_en.pdf

23. Kump PK, Krause R, Allerberger F, Högenauer C. Faecal microbiota transplantation--the Austrian approach. *Clin Microbiol Infect*. Nov 2014;20(11):1106-11. doi:10.1111/1469-0691.12801
24. Paramsothy S, Kamm MA, Kaakoush NO, et al. Multidonor intensive faecal microbiota transplantation for active ulcerative colitis: a randomised placebo-controlled trial. *Lancet (London, England)*. Mar 25 2017;389(10075):1218-1228. doi:10.1016/s0140-6736(17)30182-4
25. Costello SP, Hughes PA, Waters O, et al. Effect of Fecal Microbiota Transplantation on 8-Week Remission in Patients With Ulcerative Colitis: A Randomized Clinical Trial. *Jama*. Jan 15 2019;321(2):156-164. doi:10.1001/jama.2018.20046
26. Ni J, Shen TD, Chen EZ, et al. A role for bacterial urease in gut dysbiosis and Crohn's disease. *Sci Transl Med*. Nov 15 2017;9(416)doi:10.1126/scitranslmed.aah6888
27. Wang Y, Wiesnoski DH, Helmink BA, et al. Fecal microbiota transplantation for refractory immune checkpoint inhibitor-associated colitis. *Nat Med*. Dec 2018;24(12):1804-1808. doi:10.1038/s41591-018-0238-9
28. Fasanello MK, Robillard KT, Boland PM, Bain AJ, Kanehira K. Use of Fecal Microbial Transplantation for Immune Checkpoint Inhibitor Colitis. *ACG Case Rep J*. Apr 2020;7(4):e00360. doi:10.14309/crj.0000000000000360
29. Baumgartner M, Lang M, Holley H, et al. Mucosal Biofilms Are an Endoscopic Feature of Irritable Bowel Syndrome and Ulcerative Colitis. *Gastroenterology*. Oct 2021;161(4):1245-1256.e20. doi:10.1053/j.gastro.2021.06.024

15 TABLES AND FIGURES

Table 1 Visit and assessment schedule.

Table 2 Main efficacy results from the global, randomized, open-label phase III study (IMbrave 150) of atezolizumab plus bevacizumab versus sorafenib in patients with unresectable hepatocellular carcinoma.

Table 3 Atezolizumab infusion details.

Table 4 Bevacizumab infusion details.

Table 5 Targets and methods of stool analyses.

Table 6 Grading scale for assessing severity of adverse events that are not specifically listed in the NCI CTCAE (v5.0). Based on the most recent version of NCI CTCAE (v5.0), which can be found at: http://ctep.cancer.gov/protocolDevelopment/electronic_applications/ctc.htm

Table 7 List and explanation of exploratory objectives.

Figure 1 Overall survival in patients treated with atezolizumab plus bevacizumab achieving confirmed complete (CR)/partial response (PR) vs. stable disease (SD) vs. progressive disease (PD) using 4-month landmarks (from Ducreux M et al. Abstract # 4071, presented at ASCO Annual Meeting 2021).

Figure 2 Study schema.
